# Expanding the Usage of Lignin in DLP 3D Printing by Optimized Synthesis and Processing Parameters

**DOI:** 10.1021/acsapm.5c02394

**Published:** 2025-11-13

**Authors:** Michelle Vigogne, Anika Kaufmann, Evgeny Grigoryev, Cosima Aeschbach, Henri Lila, Michael Schwidder, Julian Thiele

**Affiliations:** † Institute of Physical Chemistry and Polymer Physics, 28408 Leibniz Institute of Polymer Research Dresden, 01069 Dresden, Germany; ‡ Institute of Chemistry, 9376 Otto von Guericke University Magdeburg, 39106 Magdeburg, Germany

**Keywords:** DLP 3D printing, high-resolution 3D printing, lignin-based resins, renewable materials, photopolymers

## Abstract

Digital light processing (DLP) has gained substantial interest in recent years as a versatile additive manufacturing technique across different disciplines. However, many materials used in DLP 3D printing are from non-sustainable sources. The use of renewable resources like lignin, a complex aromatic polymer derived from plant biomass and an industrial byproduct produced in ton-scale, in resins promotes the development of sustainable and environmentally friendly additive manufacturing processes and applications. Therefore, we first present a cost-effective and reproducible acrylation synthesis route that enables the modification of lignin using acryloyl chloride as a functionalization reagent. Further, we systematically investigate the effects of different contents of unmodified lignin up to 20 wt % and modified lignin with acrylate groups up to 30 wt % in resins using a time-efficient, self-developed step test to achieve optimal printing parameters with the smallest possible feature size. These parameters are used for high-resolution DLP 3D printing of microneedles for potential medical applications, which is why the cytotoxicity of the lignin resins is determined as well. The addition of lignin to resins influences their rheological properties and printability remarkably as well as the mechanical performance of the corresponding 3D-printed objects. Notably, higher lignin concentrations are found to enhance the mechanical strength of 3D-printed parts. Furthermore, we assess the effects of lignin acrylation in photopolymer formulations leading to improved solubility, substantial change in rheological properties from thixotropic to non-thixotropic behavior, and an enhanced E-modulus of 3D-printed materials. As a result, we showcase the feasibility to 3D-print with up to 30 wt % of modified lignin and present a 3D-printed material with 15 wt % of modified lignin, which is classified as non-cytotoxic according to EN ISO 10993-5. However, further increasing lignin content generally adversely affects the printability due to increased resin viscosity as well as light scattering and pronounced UV light absorption.

## Graphical abstract

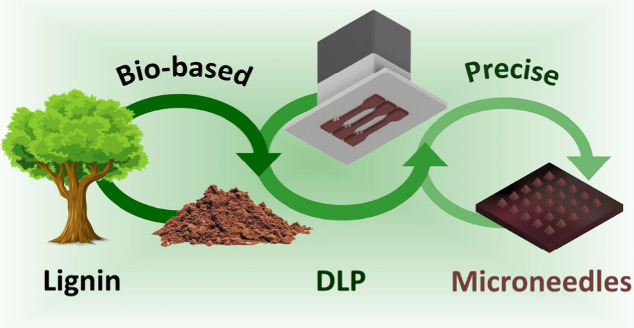

## Introduction

Additive manufacturing is considered a key technology for energy- and material-efficient production of functional material systems in small batches.^1^ In particular, it enables a tool-free production of components with complex geometric features.^2,3^ Commonly used methods for polymer processing are extrusion-based techniques (e.g., Fused Deposition Modeling, FDM) or vat polymerization like 3D printing via Digital Light Processing (DLP).^4,5^ Especially, photopolymerization processes have the advantages of less energy being required than for thermal curing techniques, a voxel-precise control of selectively exposed volume elements allows for high-resolution printing, and thermally sensitive materials can be processed without the release of volatile organic components under ambient conditions.^6,7^ Current material development for DLP 3D printing aims at moving from petrochemistry-based to renewable raw materials to achieve CO_2_ neutrality.^7^ These materials have gained much attention in additive manufacturing over recent years,^8^ and even include vegetable oils,^9^ e.g., derived from soybeans.^9–12^ In addition to the utilization of renewable sources, new material formulations should not compete with food production. One approach is to use industrial waste such as waste cooking oil^13,14^ or waste from pulp and paper processes, with lignin being its most commonly available byproduct.^7,15,16^ Approximately 20% to 30% of the dry matter in wood plants consist of lignin, with the total production of lignin by plants estimated to be approximately at 20 billion tons per year.^17^ Thus, the valorization of lignin for material development has become a clear trend, also considering that over 50 million tons of lignin are produced worldwide every year as a byproduct of biorefineries, of which only 2% is reused while 98% is used for energy production.^18,19^

There are two options for incorporating lignin in resins for DLP 3D printing: introducing unmodified lignin as filler material or using lignin modified with polymerizable groups as an active macromer for photopolymerization. In case of unmodified lignin, small amounts of lignin were reported for DLP 3D printing, e.g., unmodified softwood kraft lignin was added at up to 1 wt % to methacrylate resins^20^ or, in another example, lignin of up to 4 wt % was added to a mixture of 2-phenoxyethyl acrylate (EGPEA) as the monomer and 1,6-bis­(acryloyloxy)­hexane (HDDA) as the crosslinker.^21^ In the case of modified lignin, organosolv lignin prefunctionalized by reaction with methacrylic anhydride was used at concentrations of up to 5 wt %,^22^ up to 10% (w/v),^23^ and up to 15 wt % in a commercial resin mixture.^24^ Keck et al. presented a two-step synthesis for developing a lignin-based methacrylate resin with a lignin content between 26% and 39% that can be photopolymerized in a hot lithography process at 40 °C.^25^ In addition to its use as a macromer or filler, lignin has previously also been used as a macromolecular photoinitiator in the visible-light range for 3D printing.^26^

Apart from modifying lignin due to its photoabsorbing effect, the intensity of the light source in a DLP 3D printer is likewise crucial for achieving a maximum amount of lignin in a functional resin formulation. Considering that all studies reviewed here utilized 3D printers at an operating wavelength of 405 nm, the intensity varied from 9 mW cm^–2^ (Elegoo Mars PRO)^21^ over a laser source with an energy density of 3,167 mJ cm^–2^ and a hot lithography printer at 40 °C (Caligma 200 series, Cubicure GmbH)^25^ to a stereolithography (SLA) printer with a 120 mW Class 1 laser (Formlabs Form 1+ desktop SLA printer).^20,22,24^ These studies motivated us to systematically investigate the required light intensity in DLP 3D printing as a function of the lignin content in the resin. In addition, Böcherer et al. attempted to overcome the problem of photoabsorption by decolorizing lignin, which facilitates using up to 40 wt % lignin in DLP 3D printing.^27^ Moreover, lignin is known as a sustainable additive for mechanical reinforcement and material stiffening.^21^ Exemplarily, with unmodified softwood kraft lignin at concentrations of up to 1 wt % in a methacrylate resin, the tensile strength increased to 45–50 MPa compared to 30.7 MPa without lignin.^20^ In addition, modified lignin can substantially enhance the performance of 3D-printed objects, with a 2-fold decrease in elastic modulus and a 4-fold increase in elongation at break for 15 wt % lignin compared to the same resin made of commercial urethane-based acrylates (Genomer 1122, Ebercryl 8210) and a tetraacrylate oligomer crosslinker (SR494) without lignin.^24^ Exemplarily, we take advantage of these reports on enhanced mechanical properties by the addition of lignin and selected microneedles as an example of high-resolution DLP 3D printing with lignin, where mechanical reinforcement of the lignin-based microneedles could be beneficial for sufficient skin penetration.

## Experimental Section

### Materials

All chemicals were used without further purification unless stated otherwise. 2-Phenoxyethyl acrylate (EGPEA), 1,6-bis­(acryloyloxy)­hexane (HDDA, >93%), acrylic anhydride (>95%, stabilized by MEHQ), and *N*-hydroxy-5-norbornene-2,3-dicarboximide (NHND) were purchased from Tokyo Chemical Industries (Zwijndrecht, Belgium). Phenylbis­(2,4,6-trimethylbenzoyl)-phosphine oxide (BAPO) was obtained from BASF SE. Sulfuric acid (≥95%) and propan-2-ol (IPA, ≥99.8%) were ordered from Fisher Chemical. Dimethylformamide (DMF) was purchased from VWR Chemicals (Darmstadt, Germany). Sodium bicarbonate (≥99.5%), pyridine, deuterated chloroform, and 2-chloro-4,4,5,5-tetramethyl-1,3–2-dioxaphospholane (TMDP) were purchased from Sigma-Aldrich (St. Louis, MO, US). Acryloyl chloride (≥97%, containing approximately 400 ppm of phenothiazine as stabilizer) was purchased from Sigma-Aldrich (St. Louis, MO, US) and purified by distillation (bp 74 °C at atm. pressure). Acrylic acid (stabilized with hydroquinone monomethyl ether) for synthesis was purchased from Sigma-Aldrich and purified through Al_2_O_3_ column before use. Organosolv lignin was provided by the Fraunhofer Center for Chemical-Biotechnological Processes (Leuna, Germany). It was obtained by an ethanol–water organosolv digestion of beech wood in a lignocellulose biorefinery pilot plant (>99 purity, 90% lignin fraction with a molecular mass of 3 to 8 kDa).^28,29^

### Lignin Acrylation

5 g of lignin (23.7 mmol OH groups, determined by ^31^P NMR) were dissolved in 25 mL of DMF. The solution was heated to 60 °C, and 3.8 mL of acrylic anhydride, 2.7 mL of acryloyl chloride, or 2.4 mL of acrylic acid (1.4 times excess to OH groups in lignin) were slowly added to the solution. In the case of acrylic acid, three droplets of concentrated H_2_SO_4_ were then added to the reaction mixture. The mixture was stirred at 60 °C for 48 h, then cooled to room temperature and precipitated into 200 mL of Milli-Q water. Next, the resulting suspension was neutralized to pH 7 using a saturated NaHCO_3_ solution. The precipitate was filtered off, washed three times with 30 mL of Milli-Q water, and dried under vacuum at 30 °C for 48 h. The resulting product was obtained as light brown to black fine powder with 61% yield. Qualitatively, the introduction of acrylic groups was confirmed by the presence of a characteristic signal in the ^1^H NMR spectrum. ^1^H NMR (500 MHz, DMSO): δ 5.97 (dd, *J* = 17.3, 10.0 Hz, 1H), 5.77 (dd, *J* = 17.2, 3.2 Hz, 1H), 5.26 (d, *J* = 10.0 Hz, 1H) (cf. Figure S1). Quantitatively, the amounts of acrylic groups were evaluated by ^31^P NMR (cf. Table S1), as described before.^30^ Shortly, sample derivatization was performed in a pyridine/deuterated chloroform (1.6:1) mixture with NHND as an internal standard, where TMDP selectively reacts with hydroxy groups to form phosphorus-containing derivatives in a quantitative fashion, which can be characterized by specific ^31^P NMR chemical shifts.

### Preparation and 3D Printing of Resin

Lignin-containing resins (lignin formulations, LFM) consisting of 15 wt % HDDA as a crosslinker and 83 minus X wt % EGPEA as monomer were mixed with increasing amounts of unmodified lignin (LFMX) ground with mortar and pestle or lignin modified with acryloyl chloride (LFMXAA). Here, X stands for the concentration (in wt %) of lignin. For effective mixing and homogeneous dispersion of lignin, each formulation was sonicated three times for 10 min using a Q125 sonicator at 25% amplitude from Qsonica (Newton, CT, USA). Afterward, 2 wt % BAPO was added and mechanically stirred in a VV3 Vortex mixer from VWR (Radnor, PA, USA). For comparison, a resin without lignin was prepared (LFM0). The compositions of LFMs are summarized in Table 1.

**Table 1:** Chemical Composition of Investigated LFMs

	EGPEA wt %	HDDA wt %	lignin wt %	BAPO wt %
LFM0	83	15	0	2
LFM5/5AA	78	15	5	2
LFM10/10AA	73	15	10	2
LFM15/15AA	68	15	15	2
LFM20/20AA	63	15	20	2
LFM25AA	58	15	25	2
LFM30AA	53	15	30	2

For DLP 3D printing, two printers from ASIGA (Alexandria, Australia), namely, ASIGA Pico 2HD with a wavelength of 385 nm and an exposure intensity of 45 mW cm^–2^ and ASIGA MAX X with a wavelength of 405 nm and an exposure energy of 66 mW cm^–2^, were used. Both printers have a theoretical lateral resolution of 27 μm with a projection field of 1,920 × 1,080 pixels. CAD models were designed with the software Autodesk Inventor 2023 and sliced using the software ASIGA Composer 1.3 (2021), which was also used to set the printing parameters.

### Spot Test Measurements

For screening the cure depth as a function of exposure energy at two different wavelengths, spot tests were performed. First, 1.5 mL of the as-prepared resin was pipetted onto a glass slide and exposed at a constant exposure intensity with different exposure times to cover an exposure energy range from 4 to 3,000 mJ cm^–2^. For each exposure energy setting, three spots with a diameter of 4 mm were exposed, cleaned with IPA, and dried with compressed air. The resulting height of polymer layers was measured with a caliper gauge (TOOLCRAFT, 150 mm). This procedure was repeated for different exposure intensities until no stable polymer layer was formed due to insufficient exposure energy.

### Step Tests

A four stair-like test object was used to determine process parameters for DLP 3D printing.^31^ Each stair consisted of six steps, which differed in their exposure energy by using a constant exposure intensity (*I*) per print and different exposure times (*t*), here *t* = 2–12 s or *t* = 5–30 s or *t* = 10–60 s. By printing these six steps with different layer thicknesses (*z*) between 25 and 100 μm, the step test enabled the testing of 24 parameter combinations in one print. For a first assessment of the print resolution, a gap of 400 μm was integrated between each stair, which was used to additionally evaluate the print result. The range for the exposure energy was studied from 4 mJ cm^–2^ (2 s at 2 mW cm^–2^) to 3,600 mJ cm^–2^ (60 s at 60 mW cm^–2^) (Table S2), which corresponded to the maximum exposure intensity of the printer and provided a realistic exposure time range per layer.

### Sample Characterization

#### Rheological Measurements

Viscosity measurements of resin formulations were conducted on a Physica MCR 301 rheometer (Anton Paar GmbH, Austria) using parallel plates (*d* = 25 mm) with a measuring gap of 1 mm at 20 and 40 °C, and shear rates from 1 to 100 s^–1^.

#### Dynamic Contact Angle Measurements

Surface wettability of lignin-containing materials was studied by an optical contact angle instrument, an OCA40 (DataPhysics Instruments GmbH, Filderstadt, Germany). Dynamic contact angle measurements with sessile drop experiments were performed to determine the advancing angles (θ_adv_). For droplet formation, deionized water was used with a resistance of 18.2 MΩ cm prepared in a Milli-Q Direct 8 water purification system. The cannula of the syringe was placed in the droplet during dynamic contact angle measurement. The shape of the advancing droplet was analyzed by using the SCA 20 software (DataPhysics Instruments), and the corresponding contact angle was calculated.

#### UV/Vis Measurements

Lignin solutions were prepared in DMSO with a concentration of 1 mg mL^–1^ by shaking at 900 rpm for 2 h at 30 °C. Absorption measurements were conducted with concentrations of 0.1 mg mL^–1^ on an infinite M200 PRO plate reader (Tecan Trading AG, Switzerland) in a wavelength range from 230 to 500 nm and a step size of 1 nm.

#### Tensile Measurements

For mechanical measurements, tensile test dog bones according to DIN EN ISO 53504-S3a with different lignin contents were 3D-printed using the ASIGA MAX X at 405 nm. First, a dog-bone-shaped baseplate with 3 × 50 μm layers (0.15 mm) made of LFM0 was exposed for 1 s with 10 mW cm^–2^ to achieve good adhesion to the print head. Afterward, 74 × 25 μm layers (1.85 mm) were 3D-printed with different LFMs with exposure energies ranging from 160 to 3,600 mJ cm^–2^ (Table S3). The resulting dog bones with a final height of 2 mm were rinsed with IPA and post-cured by UV light for 5 min (UV lamp, type DR-301C, 36 W, 365 nm, 7.5 mW cm^–2^). Tensile tests compliant with DIN EN ISO527-2/S3a with an optical strain gauge were carried out with a tensile test machine (ZwickRoell GmbH & Co. KG, Ulm, Germany) with a 1 kN load cell, starting with a 0.5 N initial load and 1 mm min^–1^ test velocity, followed by a measured strain velocity of 10 mm min^–1^. Values for the elastic modulus (*E*), elongation at break (ε), and maximum stress values (σ) were determined for LFM0, LFM5, LFM10, LFM15, LFM20, LFM20AA, LFM25AA, and LFM30AA.

#### SEM Measurements

Scanning electron microscopy (SEM) images were obtained on a NEON40EsB scanning electron microscope with a field emission cathode operated at 3 kV (Carl Zeiss Microscopy Deutschland GmbH, Oberkochen, Germany) using an Everhart-Thornley detector. For evaluating object sizes, the software Olympus Stream Desktop 2.5 (Evident Europe GmbH, Hamburg, Deutschland) was used. 3D-printed squares for lateral resolution analysis and 3D-printed microneedles (Table S4) were fixed on an aluminum sample carrier with conductive silver and coated with approximately 10 nm of carbon film. For microneedles 3D-printed with LFM30AA, an additional 5 nm of platinum coating was applied by using a SCD500 coater (Leica Microsystems GmbH, Wetzlar, Germany).

#### Cytotoxicity Tests

Tensile test dog bones according to DIN EN ISO 53504-S3a were 3D-printed using the ASIGA MAX X at 405 nm. First, a dog-bone-shaped baseplate with 3 × 50 μm layers (0.15 mm) made of LFM0 was exposed for 1 s with 10 mW cm^–2^ to achieve good adhesion to the print head. Afterward, 74 × 25 μm layers (1.85 mm) were 3D-printed with LFM15, LFM15AA, or LFM30AA with exposure energies ranging from 675 to 3,600 mJ cm^–2^ (Table S5). The resulting dog bones with a final height of 2 mm were rinsed with EtOH, IPA, and acetone and post-cured by UV light exposure for 5 min (ultraviolet lamp, type DR-301C, 36 W, 365 nm, 7.5 mW cm^–2^). After that both ends of three tensile test specimens were cut off (*m* = 333 mg per part) to collect 2 × 1 g per sample, in accordance with the required amount specified by EN ISO 10993-5. The samples were then placed in EtOH for 24 h, dried under compressed air, and transferred to sterile Petri dishes. In vitro cytotoxicity tests were performed according to EN ISO 10993-5 by the National Institute of Public Health, Centre for Laboratory Testing, Laboratory of Toxicology, Laboratory No. 1206 in Prague, accredited by Czech Accreditation Institute. 1 g of each 3D-printed component was extracted according to ISO 10993-12 in 5 mL of Dulbecco’s Minimum Essential Medium supplemented with antibiotics (PNC 100 IU mL^–1^, STM 100 μ mL^–1^) and 10% of inactivated calf serum, pH 7.2 (DMEM with serum) for 24 h at 37 °C with 100 rpm agitation. Different dilutions of the extracts were prepared in DMEM with serum and incubated with Balb/c 3T3 mouse fibroblasts for 24 h (37 °C and 5% CO_2_). Afterward, the medium was removed, the cells were stained by Neutral Red dye according to DB-ALM Protocol No. 46, and the Neutral Red uptake was measured fluorimetrically. Cytotoxicity tests were determined in duplicate per material.

#### Fourier Transform Infrared Spectroscopy (FTIR)

The measurements were carried out at three points on each sample using a TENSOR II FTIR spectrometer from Bruker Optics GmbH with a platinum ATR accessory (single-reflection cell) and OPUS 7.0 software (also from Bruker) with a resolution of 2 cm^–1^ in the spectral range from 4,000 to 600 cm^–1^. The spectra were water vapor compensated, cut off at 1,800–600 cm^–1^, and baseline corrected using OPUS 7.0 software for analysis. To determine the remaining C=C bonds at the surface, the decrease in the C=C stretching vibration between the monomer mixture and the 3D-printed components was analyzed (integral analysis from 1,650 to 1,628 cm^–1^).

#### Differential Scanning Calorimetry (DSC)

The DSC measurements were performed using a Discovery 2500 device from TA Instruments (New Castle, USA). Nitrogen was used as the purge gas at a flow rate of 50 mL min^–1^. A sample weighing 7–8 mg was placed on a perforated aluminum pan. The measurement was performed in two heating cycles from −80 to 150 °C with a heating rate of 10 °C. The second heating cycle was used for evaluation and determination of the glass transition temperature (*T*_g_), as residual moisture still evaporated in the first cycle.

## Results and Discussion

### Lignin Acrylation

Resins used in DLP 3D printing require photoreactive groups to be photo-crosslinked. Although it is possible to incorporate unmodified lignin into resins,^20,21^ comparably higher lignin contents were previously achieved with modified lignin.^22–25^ Furthermore, the stability of the 3D-printed objects would decrease with increasing lignin content since unmodified lignin could not be actively incorporated into the photo-crosslinked resin and cause delamination. Previously, methacrylic anhydride was used for lignin modification^22–24^ or derivatives of it.^32^ Due to faster curing conversions of poly­(acrylates) compared to poly­(methacrylates),^22,33^ we screened different acrylation reagents for lignin modification comparing. The amounts of functional groups were determined quantitatively by ^31^P NMR (Figure 1, cf. Table S1) and used to calculate the acrylation degree.

**Figure 1:**
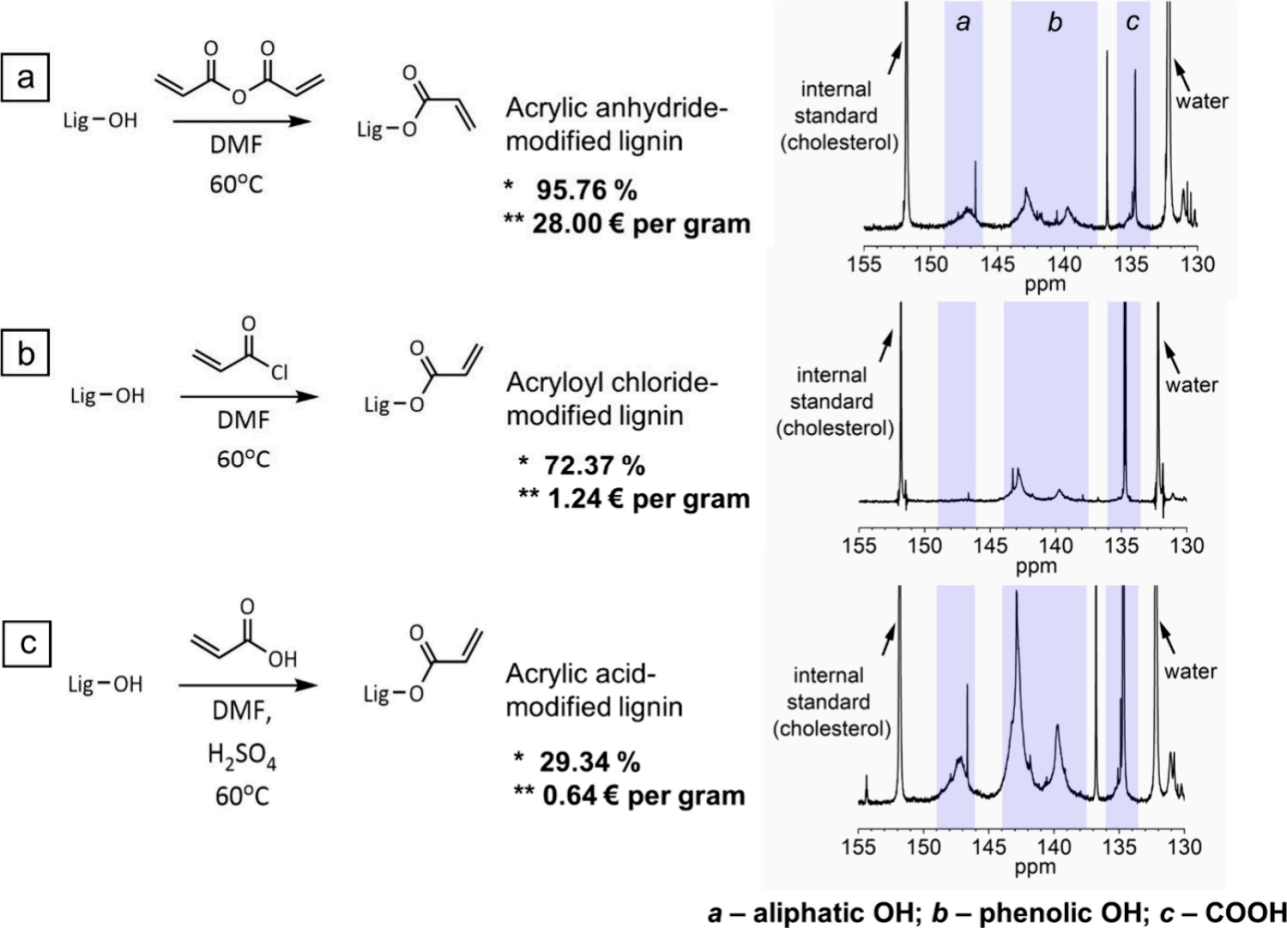
Acrylation of lignin with (a) acrylic anhydride, (b) acryloyl chloride, and (c) acrylic acid, and corresponding product analysis by ^31^P NMR. Overview of functionalization degree (*) and costs for modifying one gram of lignin (**) for different acrylation reagents. Prices are based on ordering the smallest available unit from TCI Deutschland GmbH (June 2025).

By modification of lignin with acrylic anhydride, 95.8% of the initial OH groups were modified via a S_N_ acyl reaction mechanism, with the resulting product being well-soluble in acrylate-based resin formulations. Despite good solubility of the final product, we observed notable amounts of agglomerates in the final resin formulation that we attributed to partial prepolymerization of acrylated lignin. Moreover, acrylic anhydride as acrylation reagent is cost-intensive, approximately at 28.00 € per gram of modified lignin (Figure 1a), which is a drawback for DLP 3D printing, where rather large reaction volumes in the range from 50 mL to several liters are required. Acid chlorides are known to be more reactive in S_N_ processes than acid anhydrides due to the higher relative positive charge on the carbon atom and weaker bond strength toward the heteroatom. In this regard, acryloyl chloride was tested as an acrylation reagent (Figure 1b), with which 72.4% of OH groups were converted to acrylic groups. Furthermore, the resulting product showed improved solubility depicted by the reduced number of agglomerates after mixing with an acrylate-based resin formulation, and the costs for modifying one gram of lignin were considerably lower, at 1.24 € per gram of modified lignin (Figure 1b). Lower costs, combined with a good acrylation degree and small amounts of agglomerates in the final resin formulation, make acryloyl chloride an interesting reagent for the synthesis of acrylated lignin. A further cost reduction to 0.64 € per gram of modified lignin was possible by applying acrylic acid as the acrylation reagent. In this strategy, only aliphatic OH groups of lignin were involved in the modification process, such that the maximum degree of acrylation for a batch of lignin used in this study was limited to 32.4%. Moreover, for successful modification of lignin via acrylic acid, it is also important to use a strong mineral acid as a reaction catalyst.^34,35^ This approach strongly affects the physical and chemical properties of the desired formulation, resulting in a dark lignin derivative with poor solubility in acrylate-based resin formulations. Here, the resulting degree of acrylation was 25% according to ^31^P NMR (Figure 1c). The lower acrylation degree compared to the theoretical one could be attributed to the low reactivity of acrylic acid compared to acrylic anhydride and acryloyl chloride. Moreover, the presence of strong mineral acids could cause intermolecular reactions resulting in additional chemical crosslinking of lignin, making OH groups sterically hindered for participation in the desired acrylation process.

In addition, for all acrylation agents examined here, the drying temperature of the modified lignin products appears to be a critical parameter for obtaining a finely dispersed and highly soluble acrylated lignin. Based on our observations, the optimal drying temperature is 30 °C, while an increase beyond this value can result in partial crosslinking of acrylated lignin particles, which further reduces the overall solubility.

Lignin acts as a UV-blocker which can absorb a broad spectrum of light in the range of 250 to 400 nm.^36^ This can influence the photopolymerization during DLP 3D printing, which typically takes place at 385 or 405 nm excitation wavelength. In our case, the absorption of the as-received lignin is 25% lower at a wavelength of 405 nm compared to 385 nm (Figure 2). By modifying lignin, the absorption at 405 nm further decreased by 49% for lignin modified with acrylic anhydride (acrylation degree of 95.8%) and by 18% for lignin modified with acryloyl chloride (acrylation degree of 72.4%), whereas the intrinsic absorption of lignin is not affected by modification with acrylic acid. These findings confirm earlier work by Sutton and coworkers, where acylating lignin decreased its absorption, presumably by an alteration in the electronic structure of lignin.^22^

**Figure 2:**
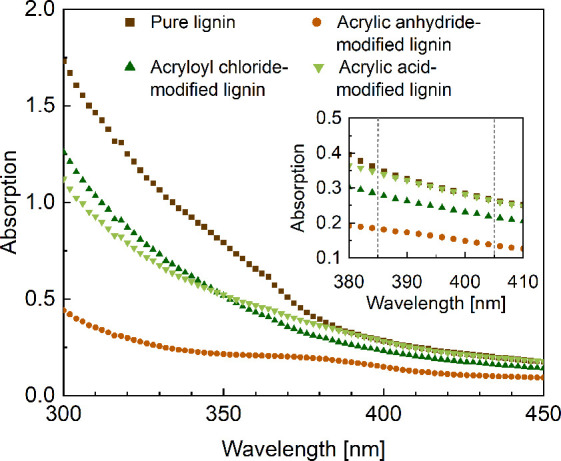
UV–Vis absorption curves of pure and chemically modified lignin (concentration 0.1 mg mL^–1^ in DMSO) with different acrylation reagents (cf. Figure 1). The inset shows differences in absorption in the range of 380 to 410 nm, whereas 385 and 405 nm are marked as the wavelengths commonly used in DLP 3D printing.

Considering the modification cost per gram of lignin, the maximum functionalization degree, and the reduction in absorbance at 405 nm, we consider the modification of lignin with acryloyl chloride to be most suitable, and we used this approach for all following experiments. In this context, the possibility of obtaining larger amounts of acrylated lignin was investigated, finally leading to a scale-up to 30 g of the target product. Theoretically, further scale-up (up to 50 or 100 g) is feasible, but may require prolonged drying time of the product, which can lead to partial oxidation of acrylic groups and crosslinking of the target product, thus reducing its solubility and reactivity.

For resin preparation with chemically modified lignin, we selected a phenolic monomer (EGPEA), which should provide improved dispersion of lignin since it also displays phenolic groups in its structure.^21^ For sufficient crosslinking of the resin during DLP 3D printing, HDDA was used. Acrylation of lignin with acryloyl chloride leads to a greatly improved dispersion of lignin in EGPEA/HDDA as proven by bright-field microscopy images showing only small agglomerates (cf. Figure S2). For unmodified lignin, an increase in agglomeration was observed from 10 wt % lignin (LFM10) to 30 wt % lignin (LFM30) with particles as large as 300–500 μm in case of resin LFM30.

### Lignin As Viscosity Enhancer

The viscosity of a resin is of technical importance in the process of layer formation in DLP 3D printing, as each layer undergoes a detachment step after polymerization, during which the cured layer peels off from the resin vat, the print head moves upward, and new resin flows into the as-formed gap. For a fast and continuous reflow, low-viscosity resins are preferable for 3D printing.^37^ Therefore, the rheological properties of resin formulations with different amounts of lignin from LFM0 to LFM50 were analyzed to evaluate their viscosity (Figure 3). The viscosity of presheared resins showed a rather constant behavior over a shear rate range from 1 to 100 s^–1^ (Figure 3a). As DLP 3D printing is a layer-by-layer vat polymerization process with low shear forces,^21,24^ viscosities at 10 s^–1^ for different lignin contents were studied in particular. Low-viscosity resins enable sufficient resin covering of the last 3D-printed layer,^38^ whereas an increasing viscosity has a negative effect on the recoating.^24^ For example, suspensions for stereolithography fabrication of ceramics should have a viscosity below 3 Pa s at 30 s^–1^ to enable sufficient recoating of the last 3D-printed layer and self-leveling in the resin vat.^39^ With increasing lignin content, higher viscosity values at a relatively low shear rate of 10 s^–1^ in the resin formulations were measured. Resin LFM10 displayed a viscosity of 0.03 Pa s at a shear rate of 10 s^–1^, which exponentially increased with an increasing lignin content up to 26.9 Pa s for LFM50 (Figure 3b). These results indicate that lignin—beyond its role in changing optical properties of a resin—also acts as effective viscosity modifier in resins. The viscosities measured at shear rates of 25 s^–1^ indicate that only resins with a lignin content of up to 40 wt % with a maximum value of 1.9 Pa s are inside the suitable viscosity range for processing via DLP 3D printing. In addition, with increasing lignin content, we observed lignin agglomerates in the resin that can be even larger than the desired layer thickness during the 3D printing process (cf. Figure S2), which is unfavorable for the printing process, as the resin vat foil can easily be damaged and the lignin particles can be detected by sensors as interfering elements. For LFM30 the particle size was investigated depending on the number of sonication cycles during resin preparation. Here, the particle size decreased with each sonication cycle; however, the viscosity behavior was not influenced (cf. Figure S2c, Figure S3).

**Figure 3:**
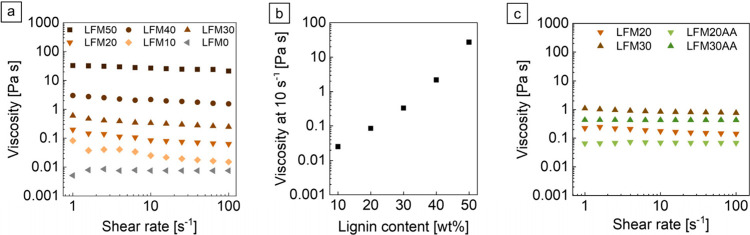
(a) Determination of viscosity of presheared LFMs in the shear rate range from 0 to 100 s^–1^, (b) correlation of viscosity at 10 s^–1^ with the amount of lignin added to the corresponding LFM, and (c) viscosity of LFM20/LFM30 and LFM20AA/LFM30AA in the shear rate range from 0 to 100 s^–1^ with preshearing for 60 s at 100 s^–1^.

Due to the high viscosity of LFM40 and LFM50, only LFM20 and LFM30 were further characterized and compared with LFM20AA and LFM30AA. After preshearing for 60 s at 100 s^–1^, the viscosity of LFM20 and LFM30 as well as of LFM20AA and LFM30AA remained stable over the entire range of tested shear rates (Figure 3c), with the overall viscosity of LFM20AA and LFM30AA being lower compared to LFM20 and LFM30. In a next step, the viscosity behavior of LFM30 and LFM30AA was measured time-dependently under low and high shear rates. When switching between a constant high shear rate of 100 s^–1^ and a low shear rate of 1 s^–1^, LFM30 showed an increase in viscosity over time (cf. Figure S4a) and therefore behaves thixotropic. Thixotropy describes the time-dependent, reversible behavior of materials whose viscosity decreases under shear and increases again after the stress has ended.^40^ Due to low shear forces during the DLP 3D printing process, the observed thixotropic behavior is undesirable, as it can lead to increased viscosity during the printing process. In contrast, the acrylation of lignin substantially changed the rheological properties of the resin, as LFM30AA showed a constant viscosity throughout the measurement time (cf. Figure S4b) and thus no thixotropic behavior. Acrylation also reduced the overall viscosity of LFM20AA and LFM30AA compared to LFM20 and LFM30 (cf. Figure 3c). This means that the acrylation of lignin has a positive effect on printability and viscosity behavior of lignin-containing resins: it improves the solubility of lignin and its compatibility with acrylate-based monomers, reduces overall resin viscosity, and provides improved resin reflow as well as recoating of the last 3D-printed layer. Further, the absence of thixotropy avoids a time-dependent viscosity gradient during the printing process. In addition, increasing the processing temperature from 20 to 40 °C could further reduce the viscosity, improving resin flow during 3D printing (cf. Figure S5).

### Influence of Lignin on Surface Wettability

Dynamic contact angle measurements were performed to analyze the influence of lignin on the surface wettability of the 3D-printed objects (Figure 4). The polymer matrix LFM0 made out of EGPEA, HDDA, and BAPO serves as a reference sample and shows hydrophobic surface properties with θ_adv_ = 95 ± 3°. The addition of lignin leads to a shift toward hydrophilic surface properties. Comparing 3D-printed objects made out of LFM20 with unmodified lignin (θ_adv_ = 69 ± 3°) with objects made out of LFM20AA with modified lignin (θ_adv_ = 65 ± 3°), no discernible differences in the hydrophilic surface properties were observed. Even a further increase in lignin in the resin did not result in a substantial lowering of the contact angle (θ_adv_ = 62 ± 3°). These results indicate that the addition of lignin with its hydrophilic hydroxyl functional groups, such as phenolic and alcoholic groups, primarily affects surface wettability. Interestingly, the transformation of hydroxyl groups to acrylate groups showed no relevant impact on wetting properties of 3D-printed objects since both functional groups have a polar character and are able to form hydrogen bonds, which indicates that the wetting behavior could be similar between the addition of unmodified and acrylated lignin due to the common hydrophilic properties.

**Figure 4:**
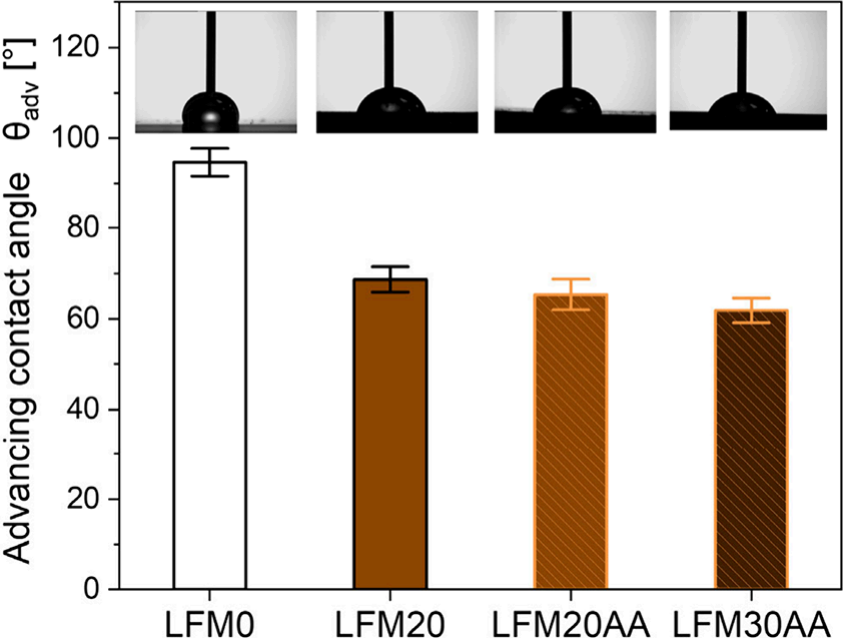
Determination of advancing contact angles to assess the surface wettability of 3D-printed objects with increasing lignin content for LFM20, LFM20AA, LFM30AA, and LFM0 as reference resin (*n* = 6 ± s.d.).

### Initial Printing Tests with Lignin-Containing Resins

First, spot tests were performed to determine important parameters for DLP 3D printing, such as the curing depth (*C*_d_), the penetration depth of the UV light (*D*_p_), and the minimum energy required to obtain a stable polymer layer (*E*_c_). For this purpose, resins were treated on a glass slide with different exposure energies, whereby the exposure intensity remained constant for one measurement. This procedure was applied to a parameter set of five exposure times. The influence of lignin content on the polymerization behavior and the process parameters can thus be analyzed. The polymerized layer thickness of a resin with different lignin content was then investigated as a function of exposure energy input (so-called Jacobs working curve, eq 1) for two wavelengths (Figure 5, cf. Table S6).^41^

1 $$C_{d}=D_{p}\ln\left(\frac{E_{\max}}{E_{c}}\right)$$

**Figure 5:**
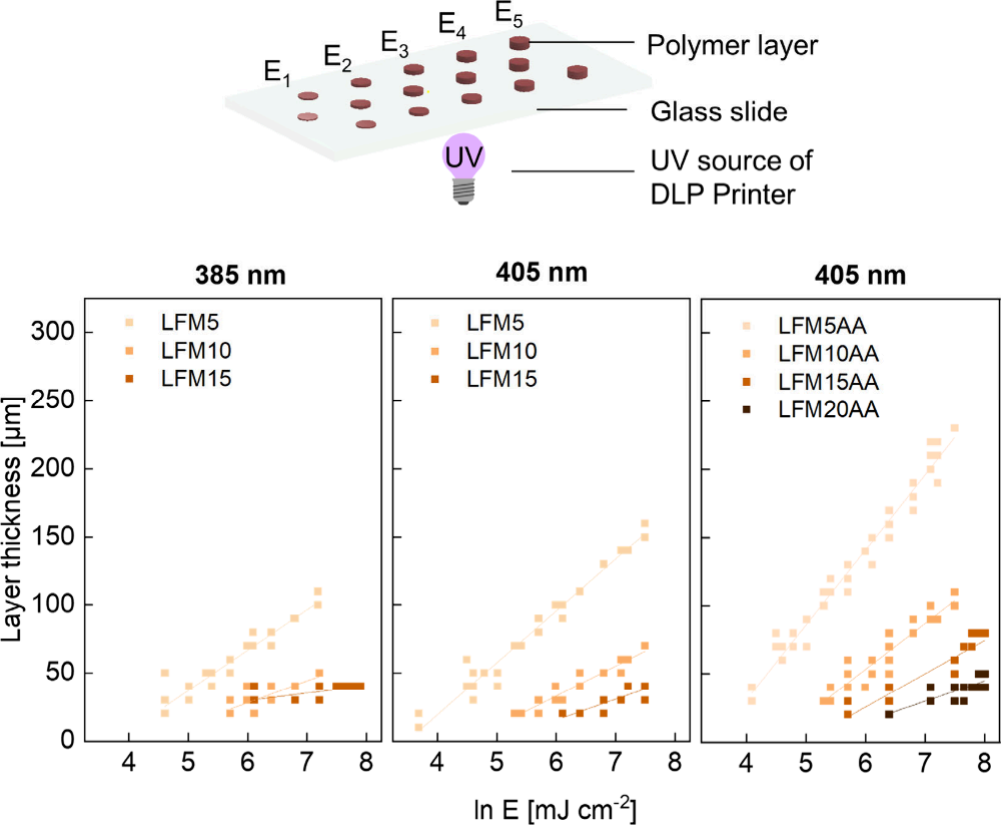
Scheme of spot tests and corresponding Jacobs working curves showing that curing depth (layer thickness) correlated to exposure energy at two different wavelengths (385 and 405 nm) for different lignin contents in a resin (here: LFM5–15, LFM5AA–20AA).

The analysis of Jacobs working curves showed that the penetration depth of UV light for LFM5 increased from 29 μm at 385 nm to 38 μm at 405 nm (cf. Table S6). Due to the lower photoabsorption, the critical energy required for photopolymerization also decreased from 42 mW cm^–2^ at 385 nm to 33 mW cm^–2^ at 405 nm for LFM5, and the resulting polymer layer thickness at an exposure energy of 900 mJ cm^–2^ rose from 90 to 130 μm. Furthermore, the use of unmodified lignin was compared to modified lignin, where the use of LFM5AA lead to an increase in layer thickness to 183 μm compared to 130 μm for LFM5. While the resulting layer thickness stagnated at less than 50 μm for LFM15, layer thicknesses of around 230 μm at 1,800 mJ cm^–2^ were realized for LFM5AA. That way, an increase of the lignin content up to 20 wt % was feasible, and polymer layers of approximately 100 μm could be realized. These results indicate the acrylation of lignin reduces the known limitations of lignin due to strong photoabsorption when acting as an active macromer. For further investigations in this work, 405 nm was chosen as the wavelength for DLP 3D printing being in a lignin spectrum range with lower photoabsorption.

### Influence of Lignin on 3D Printing Process Parameters

As DLP 3D printing is a layer-by-layer printing process, the influence of the lignin content on exposure energy in correlation to the layer height was investigated. To screen different printing parameters within one print, a staircase-shaped CAD test model was used that allows for testing six exposure times (one exposure time per staircase: *t*_1_–*t*_6_) for four different layer thicknesses (25–100 μm) at constant exposure intensity during a single print (cf. Table S7).^31^ Specifically, the exposure energies in the range of 4 to 3,600 mJ cm^–2^ were determined for layer thicknesses of 25 to 100 μm with the lignin content ranging from 0 to 30 wt % in resins (Figure 6). Thinner layer thicknesses are often preferred in DLP 3D printing to achieve accuracy and yield high-resolution prints in the micrometers range. To extend the process parameter range, the staircase-shaped objects were 3D-printed at different intensities between 2–60 mW cm^–2^ at 405 nm. The printability was judged according to a 400 μm open gap in the middle of each staircase (cf. Table S8), and the printable range with a lower (*E*_min_) and upper (*E*_max_) exposure energy limit was determined. Over- or undercured results within the tested energy range were excluded from the printable range. With increasing lignin content, *E*_min_ increased due to the photoabsorbing effect of lignin. For LFM15 to LFM20 and LFM20AA to LFM30AA, the penetration depth of the UV light and the curing depth were not sufficient to achieve 75 and 100 μm thick layers within the tested energy range, although the exposure energy was increased substantially from 1,800 to 3,600 mJ cm^–2^ to account for the increasing amount of lignin in the resin. In addition, the effects on the processability of unmodified lignin (LFMX) and modified lignin (LFMXAA) were compared. The printability of unmodified lignin was restricted to 20 wt %, which is due to the limited solubility and the increasing *E*_min_ input limited by the printer configuration. As a result of acrylation, a higher solubility was realized, and the lignin content could be increased by further 10 wt % up to LFM30AA. In particular, LFM20AA to LFM30AA showed improved processing and polymerization behavior. Thus, 25 μm layers for LFM20AA were 3D-printed in an exposure energy range from 1,200 to 2,400 mJ cm^–2^, for LFM25AA between 1,200 and 3,000 mJ cm^–2^ and for LFM30AA between 2,400 and 3,600 mJ cm^–2^. Prints with layer heights of 50 μm were achieved only with LFM20AA, but no longer with LFM25AA and LFM30AA in the tested print parameter range. Comparing these results with the working curves of Sutton et al., who used a 120 mW laser and achieved a *D*_p_ of 0.152 mm for 15 wt % lignin, it can be expected that deeper penetration depths and thus higher layer thicknesses can be achieved with higher exposure intensity.^24^ In addition to adjusting the printing process parameters, the reduced penetration depth of UV light can be useful to minimize overcuring in *z*-direction, which is particularly relevant for printing high-resolution structures (cf. Figure 7).

**Figure 6:**
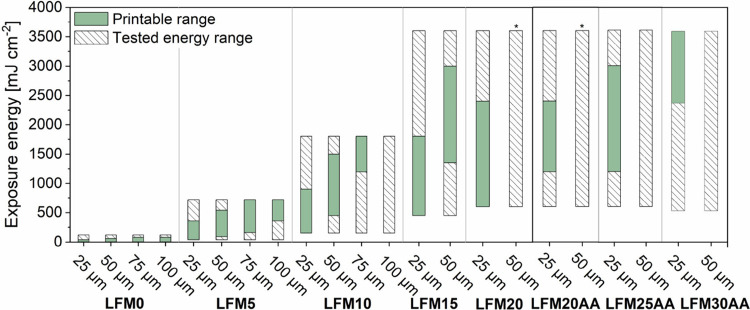
Determination of the printable range of lignin-containing resins (LFM0–LFM20 and LFM20AA–LFM30AA) via the step test. The potential printing range was determined in relation to exposure energy and set layer thickness (25, 50, 75, 100 μm). For LFM15–LFM20 and LFM20AA–LFM30AA prints with layer thicknesses of 75 and 100 μm were not performed. For prints with LFM20 and LFM20AA, 50 μm layers (*) were obtained with 3,600 mJ cm^–2^.

**Figure 7:**
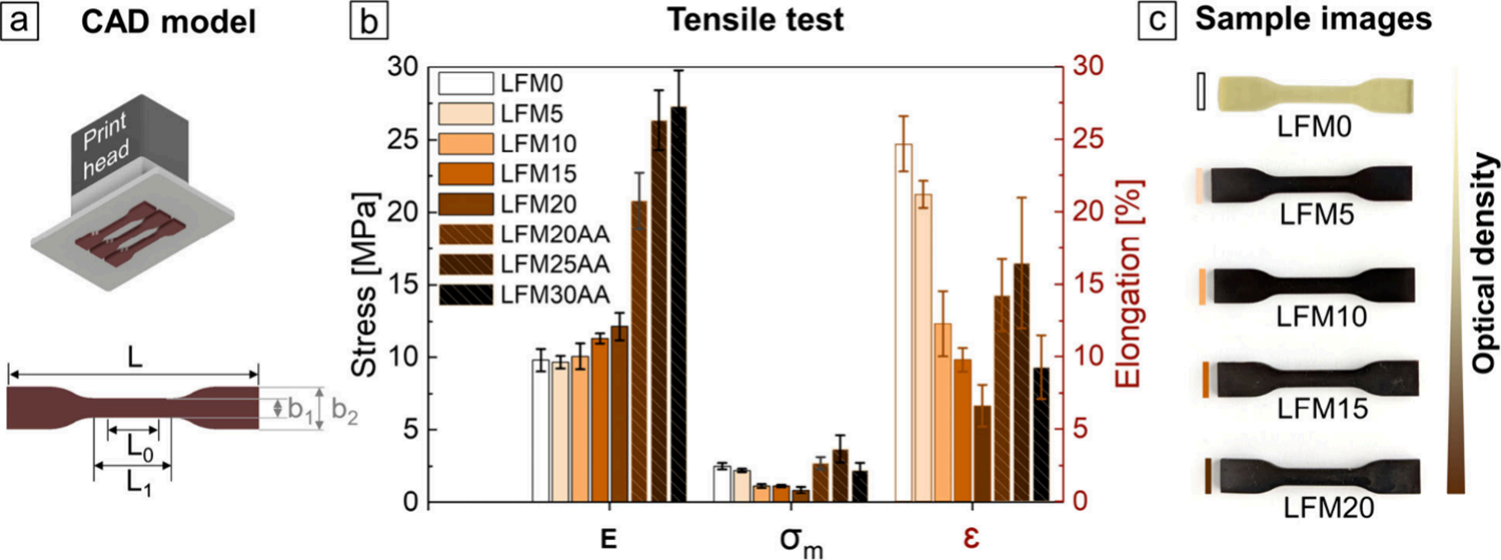
(a) CAD model of tensile test specimen type S3a according to DIN 53504 (*L* = 50 mm, *L*_0_ = 10 mm, *L*_1_ = 16 mm, *b*_1_ = 4 mm, *b*_2_ = 8.5 mm, *h* = 2 mm). (b) Evaluation of mechanical properties based on tensile testing of lignin-containing materials (LFM5–LFM20, LFM20AA–LFM30AA) compared to LFM0 including elasticity modulus, tensile strength, and elongation at break (*n* = 6 ± s.d.). (c) Optical appearance of LFM0 and lignin-containing components made from LFM5 to LFM20.

### Evaluation of Lignin As Stiffening Resin Additive

The influence of lignin content of up to 20 wt % for unmodified lignin and up to 30 wt % for modified lignin on the mechanical stability of 3D-printed components was investigated. For this purpose, tensile tests of dog bone structures according to DIN EN ISO527-2 with a modified tensile test specimen type S3a according to DIN 53504 (Figure 7a) were 3D-printed to determine mechanical properties such as elasticity modulus (*E*), tensile strength (σ_m_), and elongation at break (ε) (Figure 7b). 3D-printed dog bone structures appeared dark brown even at low lignin content (Figure 7c), resulting in an elongation of the overall printing time, due to the aforementioned photoabsorbing effect; e.g., the exposure time for printing LFM20AA was 20 s per layer, rising to 40 s for LFM25AA and to 60 s per layer for LFM30AA. Formulation LFM0 served as control and behaved like an elastic and flexible material. With increasing lignin content from LFM5 to LFM20, the 3D-printed material became stiffer, whereas the tensile strength decreased. The *E* modulus increased by 23% from 9.8 MPa for LFM0 to 12.1 MPa for LFM20, while the tensile strength σ decreased by 64% from 2.5 MPa for LFM0 to 0.9 MPa for LFM20. While the stretchability of the 3D-printed material was largely influenced by the material base EPGEA/HDDA, the elongation until breakage decreased substantially with increasing lignin content from 24.7% for LFM0 to as little as 6.6% for LFM20. Studying the differences in the influence of unmodified to modified lignin on material mechanics, we observed an increase in the *E* modulus and thus the stiffness of 3D-printed materials by 72% from 12.1 MPa for LFM20 to 20.8 MPa for LFM20AA due to active crosslinking of the acrylate groups in the polymer matrix. By further increasing the lignin content, the *E* modulus raised by 31% from 20.8 MPa for LFM20AA to 27.3 MPa for LFM30AA. In addition, the brittleness of these materials decreases compared to materials 3D-printed from resins merely blended with lignin, leading to an increase in the elongation at break from 6.6% for LFM20 to 14.2% for LFM20AA. Due to the high standard deviation, the influence of adding higher contents of modified lignin LFM20AA to LFM30AA on the elasticity trend is not evident. Overall, the following trend can be observed: with increasing *E* modulus, the elongation at break typically tends to decrease, as the enhanced stiffness and higher crosslinking density restrict the mobility of the polymer chains, causing the material to lose ductility and gain rigidity. In a previous study, 4 wt % lignin was used in combination with 15 wt % HDDA, 7 wt % TPO, and 74 wt % EGPEA where an *E* of 6.4 ± 0.6 MPa, σ of 1.29 ± 0.30 MPa, and ε of 30.4 ± 8.0% were measured,^21^ which is in a comparable range as our results. Sutton et al. investigated 3D-printed brittle materials with an elastic modulus of 0.65 GPa which was decreased by 43% with a lignin content of 15 wt % to 0.37 GPa, whereas the ductility increased from 1.87% elongation at break to 7.62%.^24^ The reinforcement effect of lignin therefore strongly depends on the lignin content. Another crucial factor is the post-curing step, with non-post-cured samples exhibiting lower tensile strength at the same tensile strain than post-cured samples with the same lignin concentration.^20^ Here, 3D-printed dog bone structures made of LFM0–LFM20 and LFM20AA–LFM30AA were also post-cured by a post-exposure with a UV light intensity of 7.5 mW cm^–2^ for 5 min.

In addition, LFM15 and LFM30AA, which were later used for printing microneedles, and LFM15AA as an additional reference sample, were further characterized by DSC to determine the glass transition temperature (*T*_g_) of corresponding 3D-printed samples and verify two previous findings: (1) it is known that lignin influences mechanothermal properties of polymer materials substantially^42^ and (2) shifts in *T*_g_ indicate different degrees of crosslinking density in a polymer network,^43,44^ thus adding to the decision process whether to use unmodified or modified lignin in resin development. The *T*_g_ increased markedly when functionalized lignin was used in the crosslinking of the resin formulations, with *T*_g_(LFM15AA) = 15.1 °C and *T*_g_(LFM15) = 1.6 °C for unmodified lignin blended into the resin. Since the polymer chain mobility is not strongly affected by physical interactions,^44,45^ the *T*_g_ value remains relatively low for LFM15. An additional increase of *T*_g_ was observed at a higher lignin content in LFM30AA, where *T*_g_ reached 20.1 °C (Table S9). This rise in *T*_g_ reflects the formation of covalent bonds, which restrict the mobility of the polymer chains through effective crosslinking.^42–44,46^ Overall, these findings confirm the results of corresponding tensile tests, where the *E* modulus was altered only to a minor degree upon addition of unmodified lignin, whereas a remarkable change was observed when using modified lignin (cf. Figure 7).

### Analysis of Lateral Resolution and High-Resolution Printing of Microneedles

The lateral resolution of 3D-printed objects is crucial for the fabrication of high-resolution structures. It is influenced by the process parameters and the reactivity of the resin. High-resolution prints were investigated with 15 wt % unmodified lignin (LFM15), as poor reproducibility was achieved for 20 wt % unmodified lignin. To analyze the lateral print resolution, pixel-aligned squares with *x*,*y*-dimensions corresponding to a multiple of the number of pixels (*N* = 4–10, pixel size = 27 μm) were 3D-printed with LFM15, and the dimensions of the CAD model were compared with the actual 3D print (Figure 8a). The analysis showed a printing accuracy in *x*-dimension of 1.19 for 3D-printed squares with *x* = *y* = 108 μm (*N* = 4) (cf. Table S10), which indicated a slight overcuring. With increasing square size, the printing accuracy in *x*-dimension improved from 1.05 for squares with *x* = *y* = 540 μm (*N* = 20) to an ideal value of 1.00 for squares with *x* = *y* = 1,080 μm (*N* = 40). An almost ideal value of 1.04 was also achieved for the printing accuracy in *y*-dimension for squares with *x* = *y* = 1,080 μm (*N* = 40).

**Figure 8:**
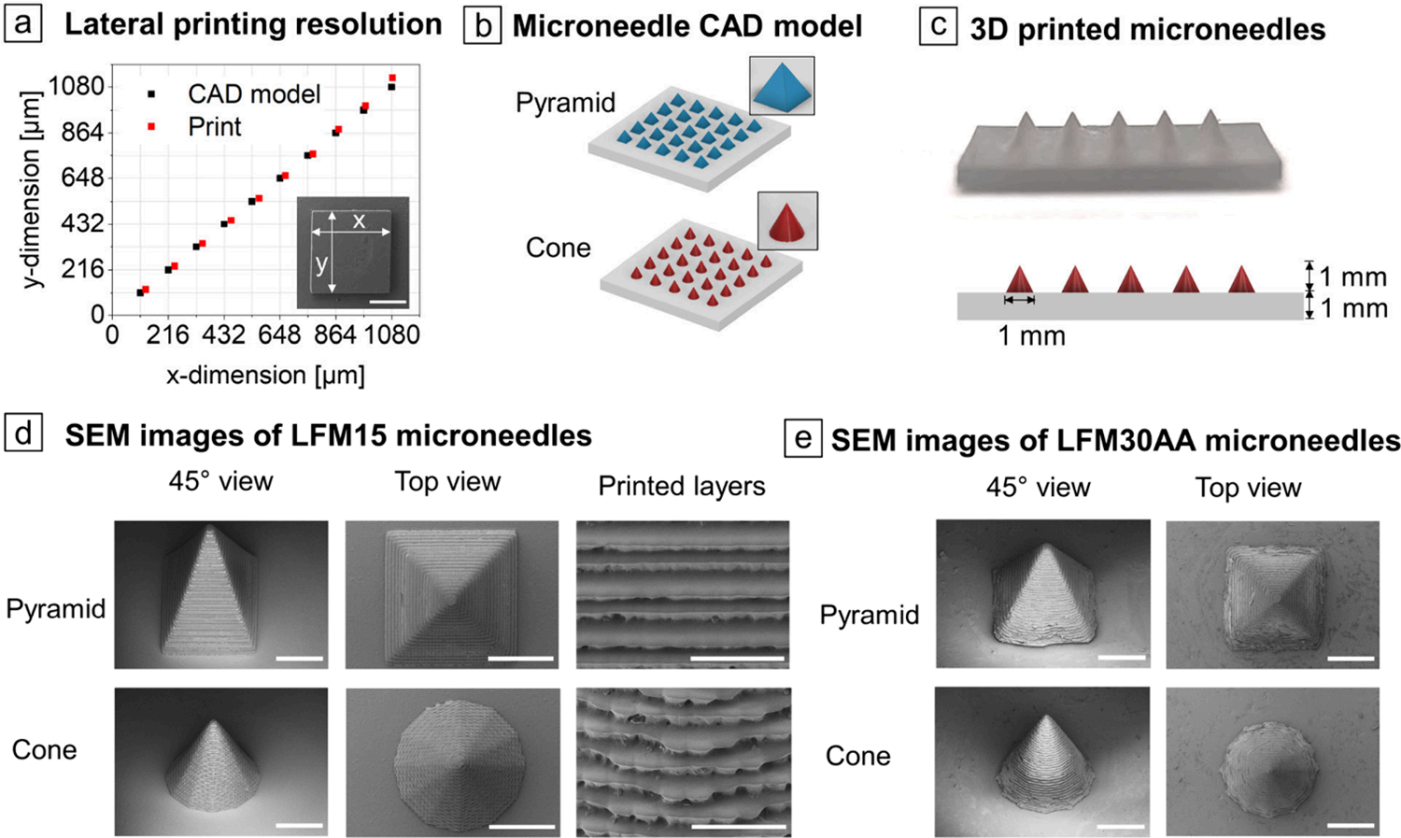
(a) Lateral dimensions (*x*-*y*) of squares 3D-printed with LFM15 compared to the CAD model. Inset: SEM image of a 3D-printed square (1,080 × 1,080 μm, scale bar indicates 500 μm). (b) CAD model of microneedles with a pyramidal and conical shape (base plate width and height is 1,000 μm). (c) Photo of the side view of 3D-printed microneedles made of LFM30AA and the corresponding CAD model. (d) SEM images of the microneedles 3D-printed with LFM15 under 45° view (scale bar indicates 500 μm) and top view (scale bar indicates 500 μm) and magnification of the 3D-printed layers under 45° view (scale bar indicates 100 μm). (e) SEM images of the microneedles 3D-printed with LFM30AA under 45° view (scale bar indicates 500 μm) and top view (scale bar indicates 500 μm).

As a proof-of-concept for the realization of high-resolution 3D-printed objects with lignin-containing resins, microneedles^47^ with a conical and a pyramidal structure and a base plate of 333 μm, 500 μm, or 1,000 μm and a height of 1,000 μm were 3D-printed (Figure 8b, 8c). The printing of microneedles with a base plate of 1,000 μm was realized with the resin LFM15 in a reliable fashion for conical and pyramidal structures and analyzed by means of SEM microscopy (Figure 8d). The size of the 3D-printed microneedles was calculated from SEM images (cf. Figure S6). For microneedles with a conical base plate of 1,000 μm, a diameter of 998 ± 1 μm and a height of 802 ± 96 μm was achieved, which relates to a printing accuracy of 1.00 and 0.80, respectively. For microneedles with a pyramidal base plate of 1,000 μm, a printing accuracy of 1.02 for the base plate and 0.80 for the height was attained (cf. Table S11). Due to the layer-by-layer printing process, the contact area of microneedles with smaller base plates was more limited than for microneedles with a larger base plate, which means that printing defects due to the presence of undissolved lignin particles or interlayer connection errors occurred more frequently and led to incomplete printing. However, for conical and pyramidal base plates of 333 and 500 μm, a printing accuracy between 1.02 and 1.10 was achieved (cf. Table S11), indicating a slight overcuring but an overall sufficient precision. The contact area between the 3D-printed layers decreased in the direction of the microneedle tip, leading to a reduced printing accuracy of 0.36 for conical base plates of 333 μm and 0.67 for 500 μm. For pyramidal base plates, a printing accuracy of 0.49 was achieved for 333 μm and 0.64 for 500 μm (cf. Table S11). Due to the improved printability, solubility, and viscosity properties of acrylated lignin, the DLP 3D printing of microneedles could be realized with 30 wt % acrylated lignin (LFM30AA). Furthermore, a better reproducibility of high-resolution prints was achieved with LFM30AA. Here, the SEM images showed a slight overcuring of the base plate (Figure 8e) resulting in printing accuracies with values from 1.03 for a conical base plate with 1,000 μm to 1.21 for a pyramidal base plate of 333 μm (cf. Table S12). Regarding the height of our microneedles, even better printing accuracy was achieved with LFM30AA than with LFM15. The values ranged from 0.57 for microneedles with a conical base plate of 333 μm, where a printing accuracy of only 0.36 was achieved for microneedles with LFM15, to 0.92 for the pyramidal base plate with 1,000 μm. Better interlayer connection could be the reason for the higher accuracy of the microneedle height when using LFM30AA instead of LFM15.

### Analysis of Biocompatibility

In addition to the sustainability aspect, the biocompatibility of the 3D-printed materials is also crucial, especially if the materials come into direct contact with the skin in later applications. For this reason, in vitro cytotoxicity tests were carried out. In this context, the used photoinitiator BAPO could be a critical component, as it is cytotoxic at higher concentrations, i.e., cell toxicity increases from 0.1–1 wt %.^48,49^ However, at lower BAPO concentrations, resins containing 30 wt % acrylated lignin could no longer be successfully 3D-printed. Furthermore, BAPO is commonly used as a photoinitiator in dentistry for photopolymerizable dental fillings and adhesives. Through the covalent bonding into the polymer network as a type I photoinitiator and high C=C conversion rate, the cell toxicity is reduced.^50^ The post-treatment of 3D-printed components is therefore important as this influences biocompatibility. For example, washing cycles with ethanol can contribute to the removal of BAPO and unreacted monomers.^51,52^ In addition, UV post-curing can be used to reduce the free acrylate groups.^52^

Consequently, the 3D-printed objects were rinsed with ethanol, isopropanol, and acetone directly after printing, then post-cured under UV light for 5 min and placed in ethanol for 24 h. A comparison of the solutions immediately after ethanol addition and after 24 h showed that unbound lignin was leached from the 3D-printed objects, especially for LFM15 (Figure S7a), which was confirmed by absorption measurements (Figure S7b). When acrylated lignin was used, considerably less lignin was washed out, with slightly more lignin being leached out of 3D-printed objects made of LFM30AA than of LFM15AA. Since the post-treatment process was suitable for dissolving unbound lignin, the 3D-printed objects washed with ethanol for 24 h were used for cytotoxicity tests.

Cytotoxicity tests according to EN ISO 10993-5 were performed using extracts of the 3D-printed objects and various dilutions thereof with Balb/c 3T3 mouse fibroblasts. Considering all extract dilutions, LFM15AA is non-cytotoxic, LFM30AA is mildly cytotoxic, and LFM15 shows severe cytotoxicity (Figure 9). Reasons for this behavior could be that the crosslinking of LFM15AA resins is more effective than that of LFM15. As a result, LFM15 has more free acrylate groups that contribute to cytotoxicity. For LFM30AA, this resin contains a larger amount of lignin compared to LFM15AA, which stabilizes radicals formed during the photopolymerization process within the phenolic units,^21^ rendering them unavailable for crosslinking and eventually causing a lower degree of polymerization of LFM30AA than LFM15AA. These assumptions are verified by FTIR measurements of the monomer mixture in comparison to 3D-printed components. Based on the C=C stretching vibration at 1,635 cm^–1^ and the C=C derformation vibration at 809 cm^–1^, it can be determined that there are still residual double bonds on the surface of the 3D-printed parts (cf. Figure S8). The integral analysis of the peak at 1,635 cm^–1^ indicates that LFM15AA has the lowest amount of residual double bonds on the surface with 5.3% of unreacted acrylate groups of the monomer or crosslinker, whereas LFM15 and LFM30 show approximately three times higher amounts of double bonds with 15.9% and 14.5%, respectively (cf. Table S13). In addition, Menima-Medzogo et al. studied the cytotoxicity of organosolv lignins from Fraunhofer CBP on human cells such as keratinocytes—a cell type from the epidermis—using a WST-1 assay and showed that it is non-cytotoxic at low concentrations of 0.2–0.4 mg mL^–1^, but cell viability decreased with increasing organosolv lignin concentration in solution.^53^ Due to different cell lines and different test methods, these results are not directly comparable. However, it can be estimated that leaching of non-crosslinked lignin in solution can also lead to a reduction in cell viability. The use of acrylated lignin is therefore advantageous since it results in active crosslinking compared to blending with unmodified lignin.

**Figure 9:**
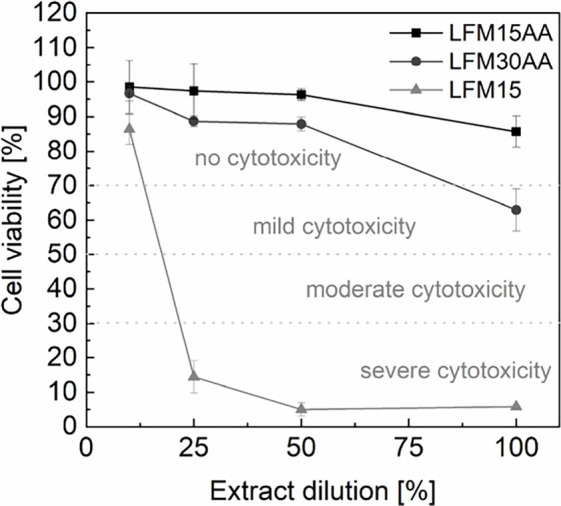
Cell viability (% of cell control, DMEM with serum) according to EN ISO 10993-5 for different extract dilutions of LFM15AA, LFM30AA, and LFM15 (*n* = 2 ± s.d.).

## Conclusion

In this study, we demonstrated the potential and limitations of biomass-derived lignin as a sustainable and cost-effective component in resin formulations for DLP 3D printing. Comprehensive characterization by rheology, contact angle measurements, mechanical tests, absorption, and Jacobs working curves for different wavelengths were applied to provide an overview of using unmodified and acrylated lignin for UV-crosslinking in DLP 3D printing. In addition, a time-efficient step test was used to provide straightforward insights into process-relevant printing parameters, such as exposure energy, layer thicknesses, and printing accuracy. The use of our step test enabled rapid screening of 24 parameters per 3D print, which was successfully used to investigate different amounts of unmodified and acrylated lignin in the resins. With these tests and characterization methods, we have presented and compared two methods of using lignin: as conventional filler material, where commonly used resin base material could be blended with up to 20 wt % lignin, and chemically modified lignin with acrylate groups, which enabled a higher lignin content of up to 30 wt %. These results highlight the importance of continuous method development for rapid screening of process parameters of resins to ensure sufficient exposure energies for good printability at varying layer thicknesses, particularly when using biomass-derived materials such as lignin.

In addition, we demonstrate that chemical modification of lignin enhances the material properties: it reduces UV absorption and viscosity, eliminates thixotropic behavior of unmodified lignin, and improves polymerization behavior, particularly layer-to-layer connection in DLP 3D printing. The application of acrylated lignin in the investigated resins leads to a notable increase in mechanical strength of corresponding 3D-printed materials compared to unmodified lignin in the resin, improving stiffness and reducing brittleness. Based on these studies, high-resolution DLP 3D printing was exemplarily shown by manufacturing microneedles with 30 wt % acrylated lignin. Considering the cytotoxicity studies, LFM15AA is non-cytotoxic, while LFM30AA is moderately cytotoxic and LFM15 exhibits severe cytotoxicity. Therefore, for applications of medical microneedles with contact to skin, LFM15AA would be a good compromise between biocompatibility and replacing 15 wt % of a petroleum-based resin component with a renewable material such as lignin.

Limitations to further enhance the lignin content in resins for DLP 3D printing arise from an inappropriate viscosity increase of the resulting resin formulation beyond 30 wt % lignin, accompanied by an increased lignin particle formation and agglomeration as well as photoabsorption. Thus, thinner layers have to be 3D-printed with the risk of cracked, unstable, and fractured layers as well as layer delamination, especially in the presence of undissolved lignin particles. In addition, a higher lignin content requires longer exposure times per layer such that a longer overall printing time has to be accepted. On the other hand, these limitations can also be turned into an advantage as lignin can be discussed as sustainable photoabsorber, preventing overcuring in lateral dimensions and thus a loss in printing accuracy. In summary, these results demonstrate that lignin can replace petroleum-based components of resin formulations for DLP 3D printing, paving the way toward more sustainable and cost-effective polymer material solutions.

## Supplementary Material



## Notes

^1^H NMR of acrylated lignin; amounts of functional groups in acrylated lignin based on ^31^P NMR; bright-field microscopy images of lignin particles in EGPEA/HDDA; viscosity measurements of LFM30 for 2–4 sonication cycles; time-dependent viscosity of LFM30 and LFM30AA; temperature-dependent viscosity measurements; parameter settings for step tests; images of 3D-printed step test structures; equation for calculating microneedle height; determination of the lateral printing resolution for 3D-printed squares and for high-resolution printing of lignin-based microneedles; release of lignin from 3D-printed objects, FITR-measurements of monomer mixtures compared to 3D printed components, DSC measurements for T_g_ value determination (PDF)
