# Supplementary material for: Expanding the Usage of Lignin in DLP 3D Printing by Optimized Synthesis and Processing Parameters
Source: ACS Appl Polym Mater. 2025 Nov 13;7(22):15255–67. doi: 10.1021/acsapm.5c02394 (PMC12670356; doi:10.1021/acsapm.5c02394)
Supplement: Supplementary file 1 [file ap5c02394_si_001.pdf]

Supporting Information

Expanding the usage of lignin in DLP 3D printing by optimized synthesis and processing parameters

Michelle Vigogne[1†], Anika Kaufmann[1†,], Evgeny Grigoryev[1], Cosima Aeschbach[1], Henri Lila[1], Michael Schwidder[2], Julian Thiele[1,2,*]*

ORCID: Michelle Vigogne (0009-0009-8788-1026), Anika Kaufmann (0009-0008-6877-4316), Evgeny Grigoryev (0000-0002-0538-1864), Cosima Aeschbach (0009-0000-8246-0489), Henri Lila (0009-0002-5898-4316), Michael Schwidder (0000-0001-5098-574X), Julian Thiele (0000-0001-5449-3048).

[1] Institute of Physical Chemistry and Polymer Physics, Leibniz Institute of Polymer Research Dresden, 01069 Dresden, Germany

[2] Institute of Chemistry, Otto von Guericke University Magdeburg, 39106 Magdeburg, Germany

* kaufmann@ipfdd.de, julian.thiele@ovgu.de

[†] These authors contributed equally to this work.

Note: LFM (lignin formulations) stands for *lignin-containing resins* (cf. main manuscript).

Figures:

Figure S1. ^{1}H NMR of lignin modified with acryloyl chloride.

Figure S2. Comparison of homogeneity in resin formulations using bright-field microscopy images.

Figure S3. Determination of viscosity of resin formulation LFM30.

Figure S4. Time-dependent viscosity measurements of LFM30 and LFM30AA.

Figure S5. Temperature-dependent viscosity measurements of LFM20 and LFM30.

Figure S6. Microneedle schematics for determining actual 3D-printed height.

Figure S7. Determining the release of uncured lignin from 3D-printed objects into ethanol via absorption measurements.

Figure S8: FTIR spectra of monomer mixtures compared to DLP 3D-printed components after post-processing with UV light and 24 hours in EtOH of a) LFM15, b) LFM15AA, and c) LFM30AA.

Tables:

Table S1. Molar amounts of aliphatic OH, phenolic OH and COOH groups in (un-)modified lignin based on ^{31}P NMR data.

Table S2. Tested 3D printing parameter range depending on used LFM.

Table S3. Printing parameters for tensile tests of 3D-printed dog bones with different LFMs.

Table S4. Printing parameters for microneedle fabrication.

Table S5. Printing parameters for cytotoxicity tests with different LFMs.

Table S6. Influence of lignin content on layer thickness (d), penetration depth (D_p), and critical energy (E_c).

Table S7. Parameter settings for step test screening.

Table S8. Images of 3D-printed step tests to determine the printing parameter range of resins.

Table S9. Determination of glass transition temperature of LFM15, LFM15AA, and LFM30AA using DSC measurements.

Table S10. Determination of lateral printing resolution for 3D-printed squares made of LFM15.

Table S11. Determination of printing accuracy of microneedles using resin formulation LFM15.

Table S12. Determination of printing accuracy of microneedles using resin formulation LFM30AA.

Table S13. Determination of residual surface double bonds from FTIR spectra.

Equation:

Equation Eq. 1. Determining the printed height (h) of a microneedle.

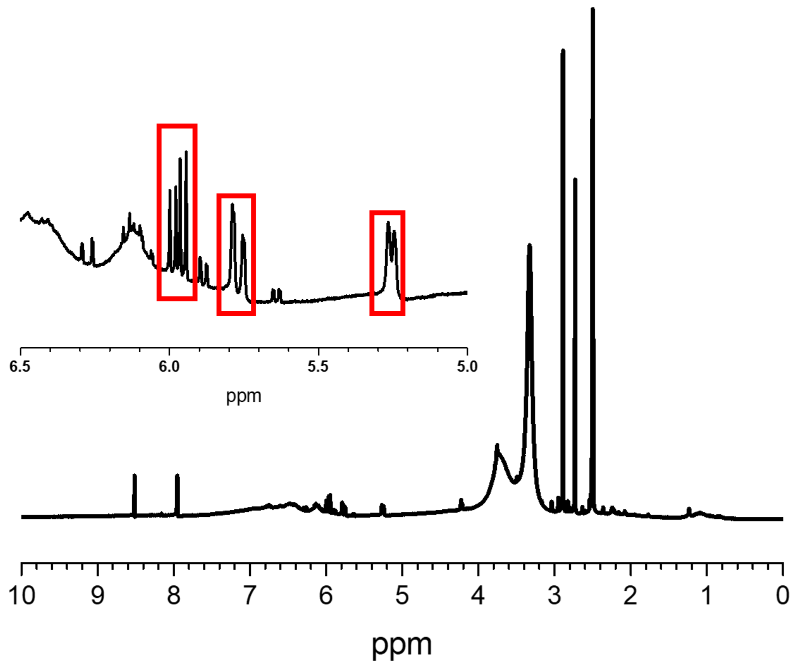

Figure S1. ^{1}H NMR of lignin modified with acryloyl chloride. Highlighted multiplets correspond to the successful incorporation of acrylic groups.

Table S1. Molar amounts of aliphatic OH, phenolic OH and COOH groups in (un-)modified lignin based on ^{31}P NMR data.

Pure lignin or acrylation reagent	Amounts of functional groups (mmol g^{-1})		
	Aliphatic OH	Phenolic OH	COOH
**Pure lignin**	1.92	3.85	0.14
**Acrylic anhydride-modified lignin**	0.05	0.15	0.05
**Acryloyl chloride--modified lignin**	0.28	0.74	0.61
**Acrylic acid--modified lignin**	0.44	2.18	1.17

Table S2. Tested 3D printing parameter range depending on used LFM.

Sample	Exposure intensity [mW cm^{-2}]	Exposure time [s]	Exposure energy [mJ cm^{-2}]
LFM0	2, 5, 10	2 - 12	4 - 120
LFM5 / 5AA	20, 30, 45, 60	2 - 12	40 - 720
LFM10 / 10AA	30, 45, 60	5 - 30	150 - 1,800
LFM15 / 15AA	45, 60	10 - 60	450 - 3,600
LFM20 / 20AA	60	10 - 60	600 - 3,600
LFM25AA	60	10 - 60	600 - 3,600
LFM30AA	60	10 - 60	600 - 3,600

Table S3. Printing parameters for tensile tests of 3D-printed dog bones with different LFMs.

Sample	Layer thickness [µm]	Exposure energy [mJ cm^{-2}]
LFM0	25	10
LFM5	25	160
LFM10	25	300
LFM15	25	675
LFM20 / 20AA	25	1,200
LFM25AA	25	2,400
LFM30AA	25	3,600

Table S4. Printing parameters for microneedle fabrication.

Sample	Layer thickness [µm]	Exposure energy [mJ cm^{-2}]
LFM15	25	1,125
LFM30AA	25	3,600

Table S5. Printing parameters for cytotoxicity tests with different LFMs.

Sample	Layer thickness [µm]	Exposure energy [mJ cm^{-2}]
LFM15	25	675
LFM15AA	25	675
LFM30AA	25	3,600

Supporting Information

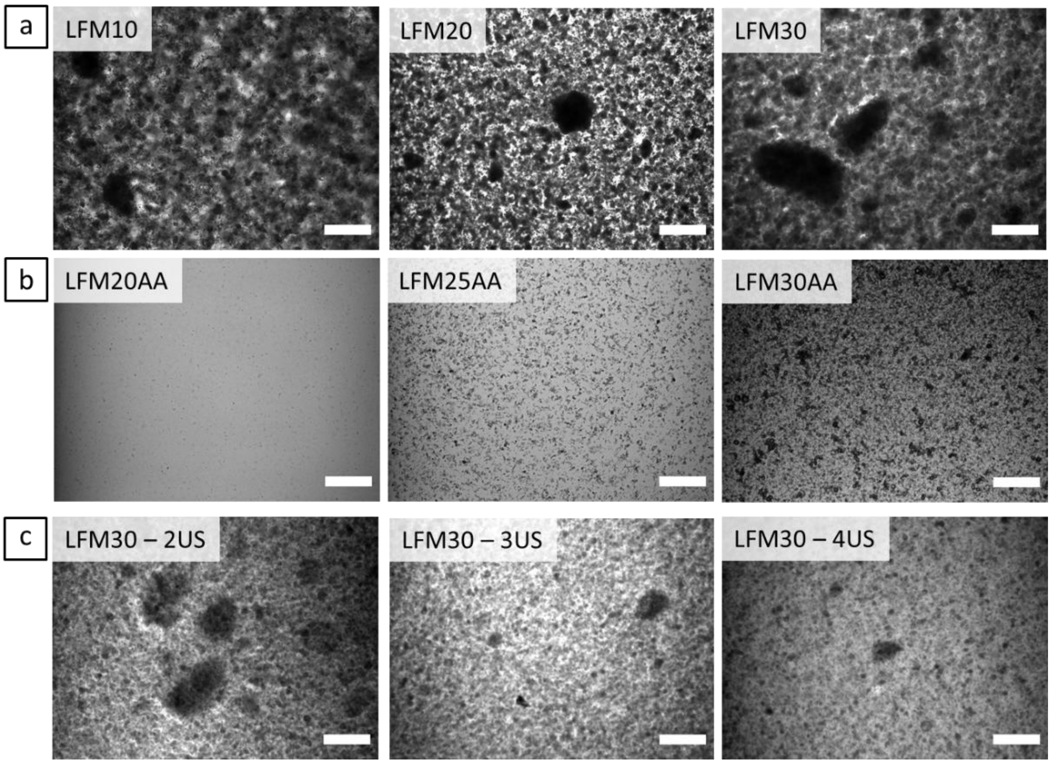

Figure S2. Comparison of homogeneity in resin formulations and occurrence as well as size of lignin particles therein. Bright-field microscopy images of a) unmodified, b) acrylated, and c) unmodified lignin after 2 to 4 sonification cycles (US) in mixtures of ethylene glycol phenyl ether acrylate and 1,6-hexanediol diacrylate (EGPEA/HDDA; resin formulation name LFM"X": 15% (w/w) HDDA, 83 to X% (w/w) EGPEA, X% (w/w) lignin, cf. Table 1 in main manuscript). Scale bars denote 200 µm.

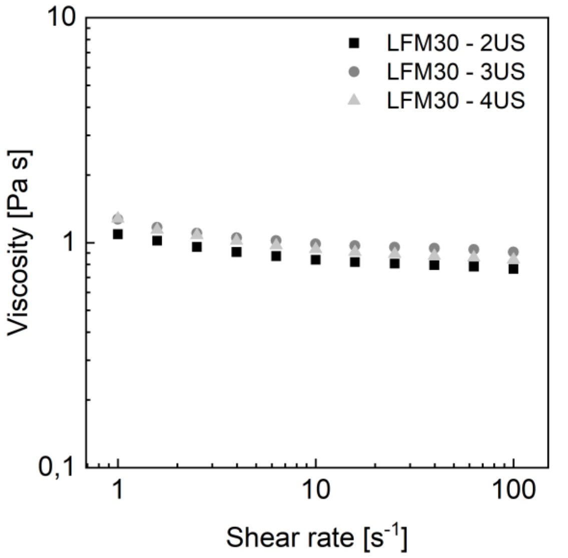

Figure S3. Determination of viscosity of resin formulation LFM30 presheared for 60 s and 100 s^{-1} containing different particle sizes after 2 - 4 sonification cycles in the shear rate range from 0 to 100 s^{-1}.

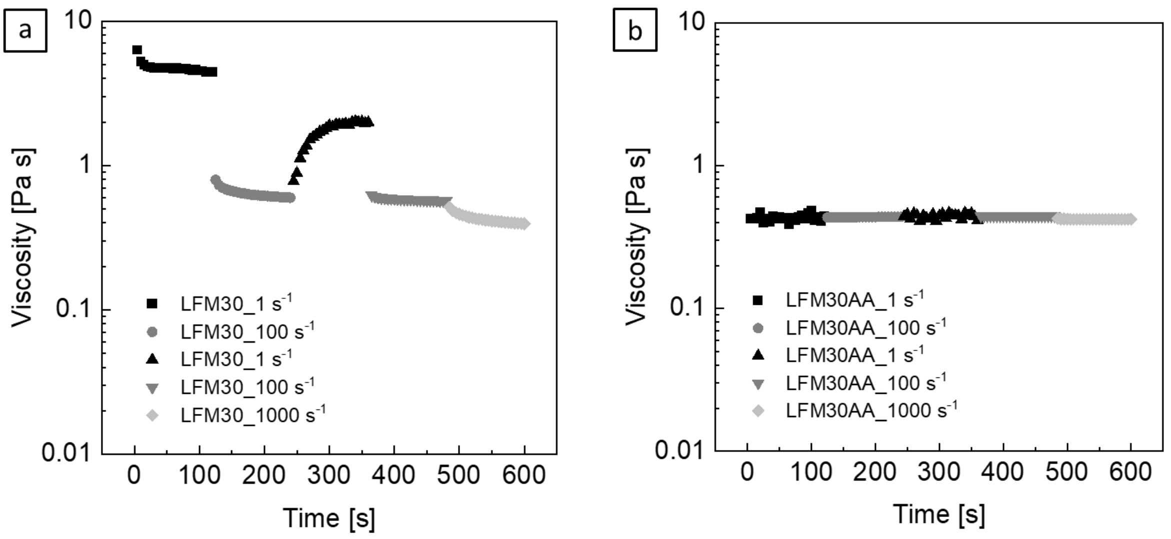

Figure S4. Time-dependent viscosity measurements at constant shear rates of 1, 100 and 1,000 s^{-1}, a) LFM30 indicating a thixotropic behavior, b) LFM30AA showing no sign of thixotropic behavior.

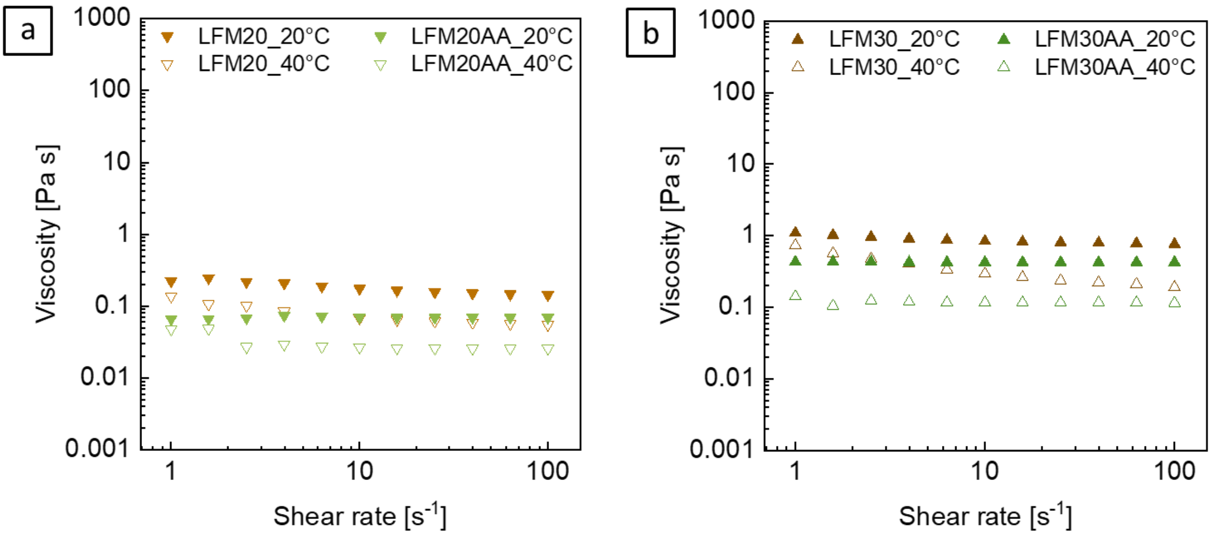

Figure S5. Temperature-dependent viscosity measurements in a shear rate range from 0 to 100 s^{-1} at 20 °C and 40 °C, a) LFM20 and LFM20AA, b) LFM30 and LFM30AA. All samples are presheared for 60 s with 100 s^{-1}.

Table S6. Influence of lignin content on layer thickness (d), penetration depth (D_p), and critical energy (E_c).

	d [µm] *	D_p [µm]	E_c [mJ cm^{-2}]
**LFM5 (λ = 385 nm)**	90	29	42
**LFM5 (λ = 405 nm)**	130	38	33
**LFM5AA (λ = 405 nm)**	183	55	31

* d at 900 mJ cm^{-2} (30 s at 30 mW cm^{-2}).

Table S7. Parameter settings for step test screening and energy range from 4 to 3,600 mJ cm^{-2}.

	t_1 [s]	t_2 [s]	t_3 [s]	t_4 [s]	t_5 [s]	t_6 [s]	I [mW cm^{-2}]
**Setting 1**	2	4	6	8	10	12	2 – 60
**Setting 2**	5	10	15	20	25	30	2 – 60
**Setting 3**	10	20	30	40	50	60	2 – 60

Table S8. Images of 3D-printed step tests to determine the printing parameter range of resins depending on their lignin content for LFM0 to LFM20, and LFM20AA to LFM30AA. The potential printing range was determined in relation to exposure energy and set layer thickness (25, 50, 75, 100 µm). For LFM15, LFM20 and LFM20AA, LFM25AA, LFM30AA, layer thicknesses of 75 µm and 100 µm were not in the printable range.

	**LFM0**<br>2 - 12 s		**LFM5**<br>2 - 12 s
2 mW	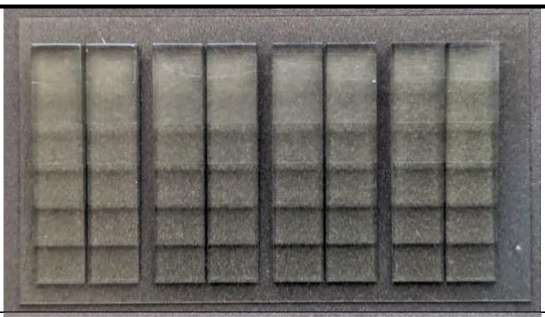	20 mW	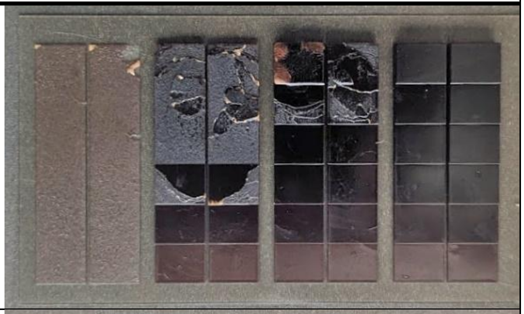
5 mW	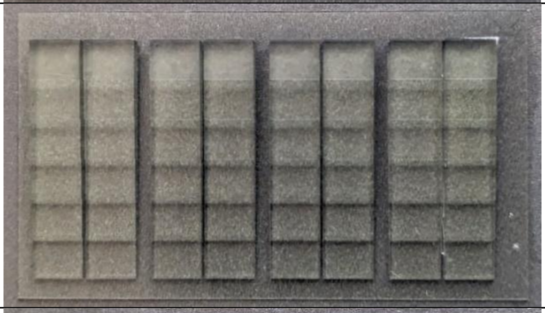	30 mW	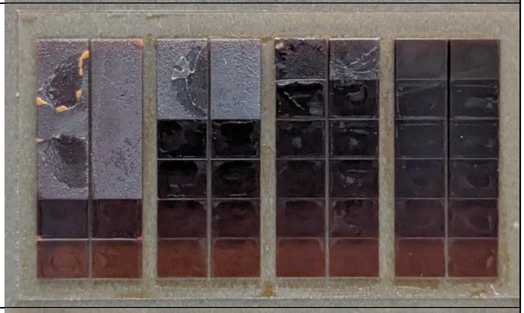
10 mW	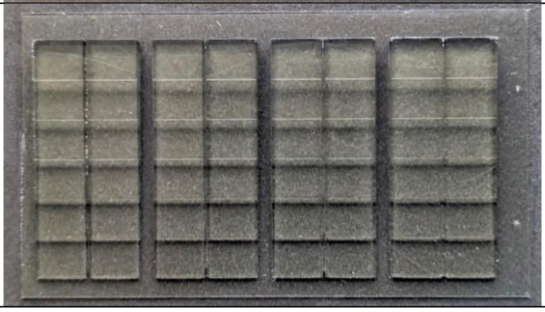	45 mW	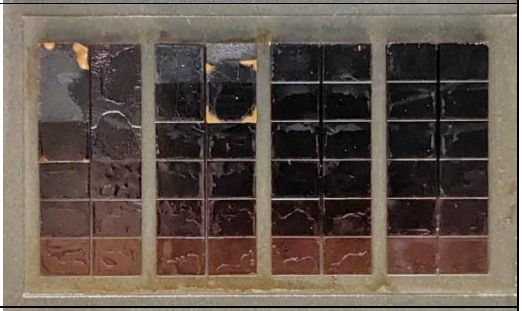
		60 mW	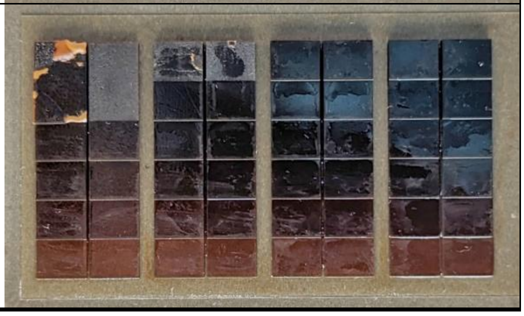

	**LFM10**<br>5 - 30 s		**LFM15**<br>10 - 60 s
30 mW	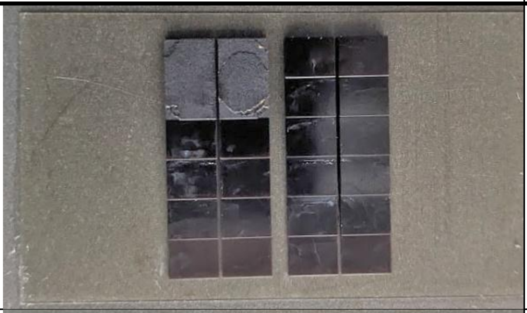		
45 mW	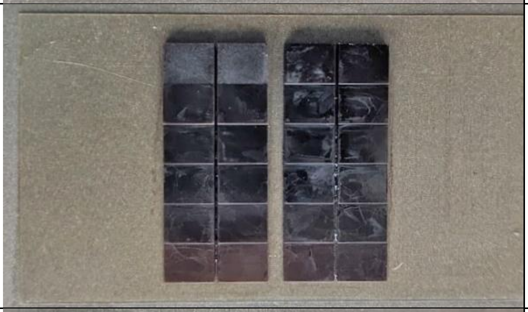	45 mW	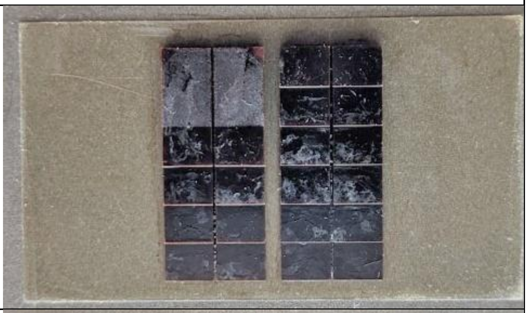
60 mW	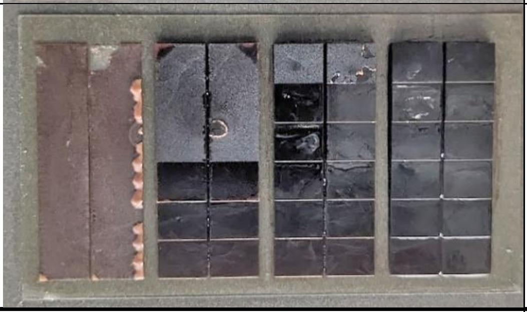	60 mW	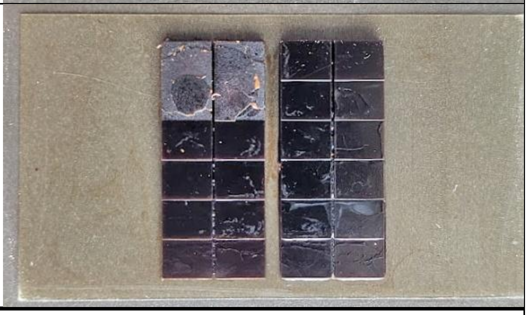
	**LFM20**<br>10 - 60 s		
60 mW	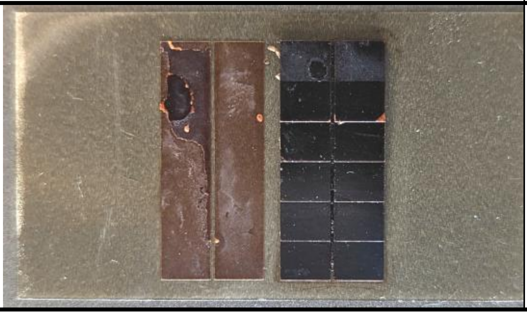		

	**LFM20AA**<br>10 - 60 s		**LFM25AA**<br>10 - 60 s
60 mW	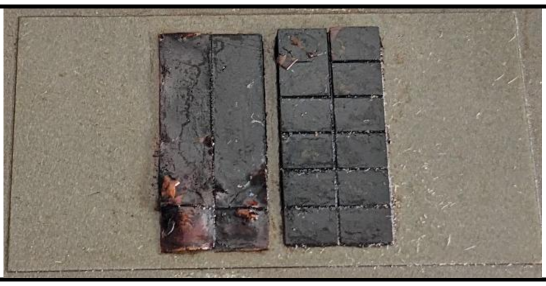		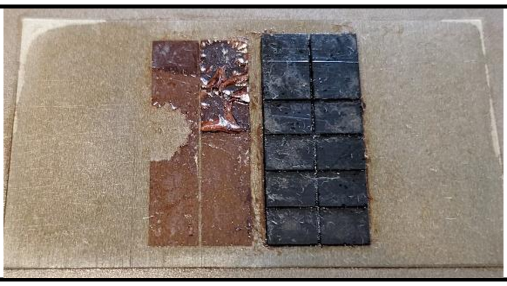
	**LFM30AA**<br>10 - 60 s		
60 mW	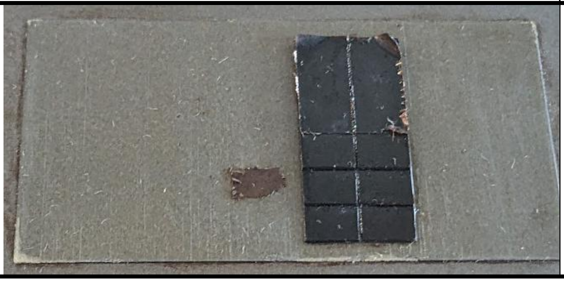		

Table S9. Determination of glass transition temperature of LFM15, LFM15AA and LFM30AA using DSC measurements.

Sample	Glass transition temperature Tg [°C]
**LFM15**	1.6
**LFM15AA**	15.1
**LFM30AA**	20.1

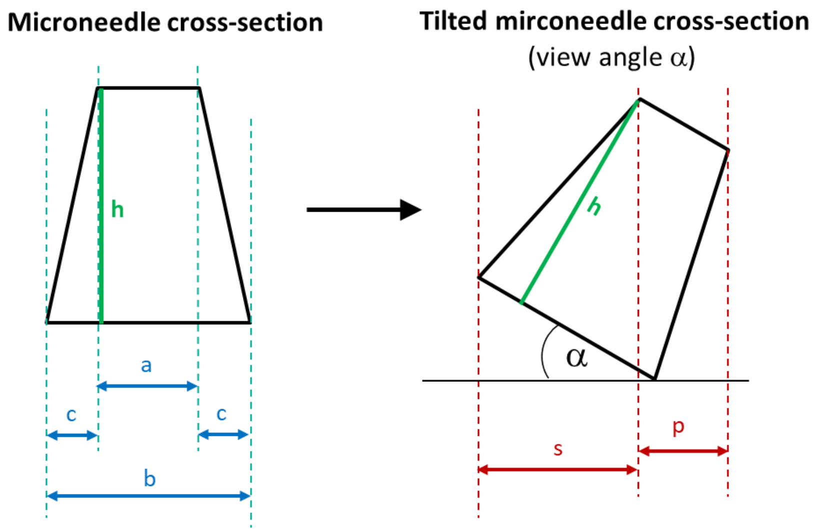

Figure S6. Microneedle schematics for determining actual 3D-printed heights (h) and base plate dimensions (b). Microneedles were characterized via SEM in the top view and under a viewing angle α = 45°. The dimensions of a, b, c were measured using SEM images, and h derived from that (cf. **Eq. 1**).

To determine the printed height (h) of a microneedle, the dimensions of a, b, c were measured from SEM images using the top view, and the distance s was measured in a tilted view. The height of an individual microneedle was calculated applying the following equation (**Eq. 1**):

$$h = \frac{s - c \cdot \cos(\alpha)}{\sin(\alpha)} \quad \textbf{(Eq. 1)}$$

Table S10. Determination of lateral printing resolution for 3D-printed squares as test objects with different pixel sizes using **resin formulation LFM15**.

	CAD model		3D-printed squares[1]		Printing accuracy[2]	
**Pixel N**	**x-dimension [µm]**	**y-dimension [µm]**	**x-dimension [µm]**	**y-dimension [µm]**	**x-dimension**	**y-dimension**
4	108	108	129 ± 2	124 ± 1	1.19	1.15
8	216	216	241 ± 2	234 ± 1	1.12	1.08
12	324	324	349 ± 2	340 ± 2	1.08	1.05
16	432	432	459 ± 3	450 ± 1	1.06	1.04
20	540	540	567 ± 1	555 ± 1	1.05	1.03
24	648	648	669 ± 1	661 ± 4	1.03	1.02
28	756	756	776 ± 3	763 ± 1	1.03	1.01
32	864	864	875 ± 3	881 ± 1	1.01	1.02
36	972	972	979 ± 1	992 ± 0	1.01	1.02
40	1,080	1,080	1,083 ± 2	1,124 ± 1	1.00	1.04

[1] mean ± s.d. (n = 3); [2] ratio of 3D-printed dimensions to CAD dimensions

Table S11. Determination of lateral resolution and printing accuracy for high-resolution 3D printing of microneedles using **resin formulation LFM15**.

	CAD model		3D-printed microneedles[1]		Printing accuracy[2]	
**No.**	**d [µm]**	**h [µm]**	**d [µm]**	**h [µm]**	**d**	**h**
**Conical base plate**						
1	333	1,000	353 ± 1	355 ± 224	1.06	0.36
2	500	1,000	510 ± 2	673 ± 96	1.02	0.67
3	1,000	1,000	998 ± 1	802 ± 96	1.00	0.80
**Pyramidal base plate**						
1	333	1,000	365 ± 2	490 ± 84	1.10	0.49
2	500	1,000	528 ± 1	640 ± 140	1.06	0.64
3	1,000	1,000	1,022 ± 2	804 ± 68	1.02	0.80

[1] mean ± s.d. (n = 3); [2] ratio of 3D-printed dimensions to CAD dimensions

Table S12. Determination of lateral resolution and printing accuracy for high-resolution printing of microneedles using **resin formulation LFM30AA**.

	CAD model		3D-printed microneedles[1]		Printing accuracy[2]	
**No.**	**d [µm]**	**h [µm]**	**d [µm]**	**h [µm]**	**d**	**h**
**Conical base plate**						
1	333	1,000	378 ± 3	571 ± 172	1.14	0.57
2	500	1,000	537 ± 11	685 ± 161	1.07	0.68
3	1,000	1,000	1,029 ± 9	894 ± 53	1.03	0.89
**Pyramidal base plate**						
1	333	1,000	402 ± 5	616 ± 282	1.21	0.62
2	500	1,000	581 ± 16	912 ± 39	1.16	0.91
3	1,000	1,000	1,106 ± 5	918 ± 59	1.11	0.92

[1] mean ± s.d. (n = 3-5); [2] ratio of 3D-printed dimensions to CAD dimensions

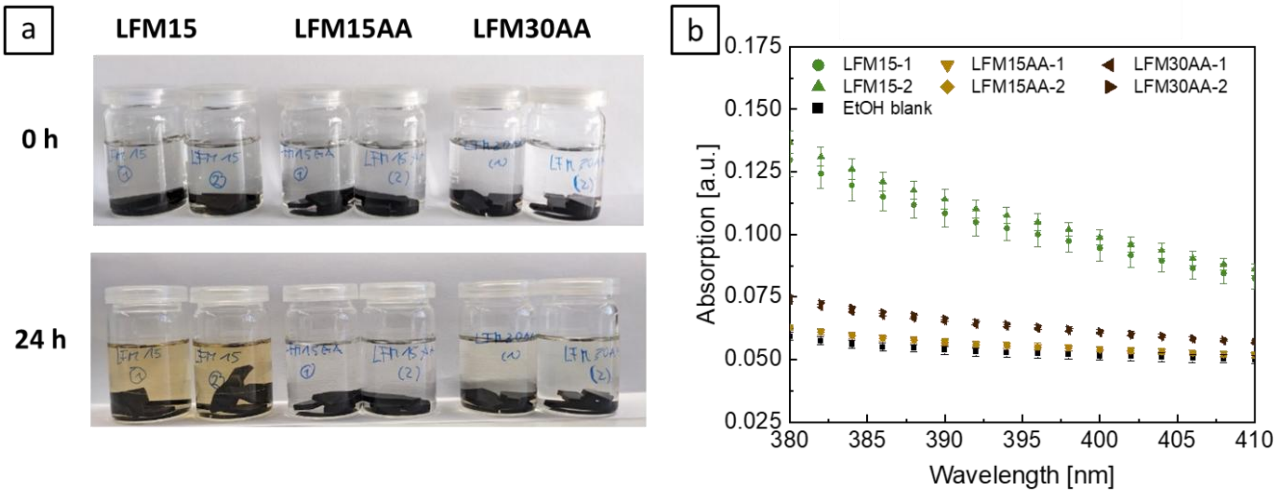

Figure S7. Determing the release of uncured lignin from 3D-printed objects made of LFM15, LFM15AA, and LFM30AA into ethanol; a) photograph of samples directly after addition of ethanol (0 h) and after 24 h; b) absorption measurements of ethanol solutions after 24 h (n = 3 ± s.d.).

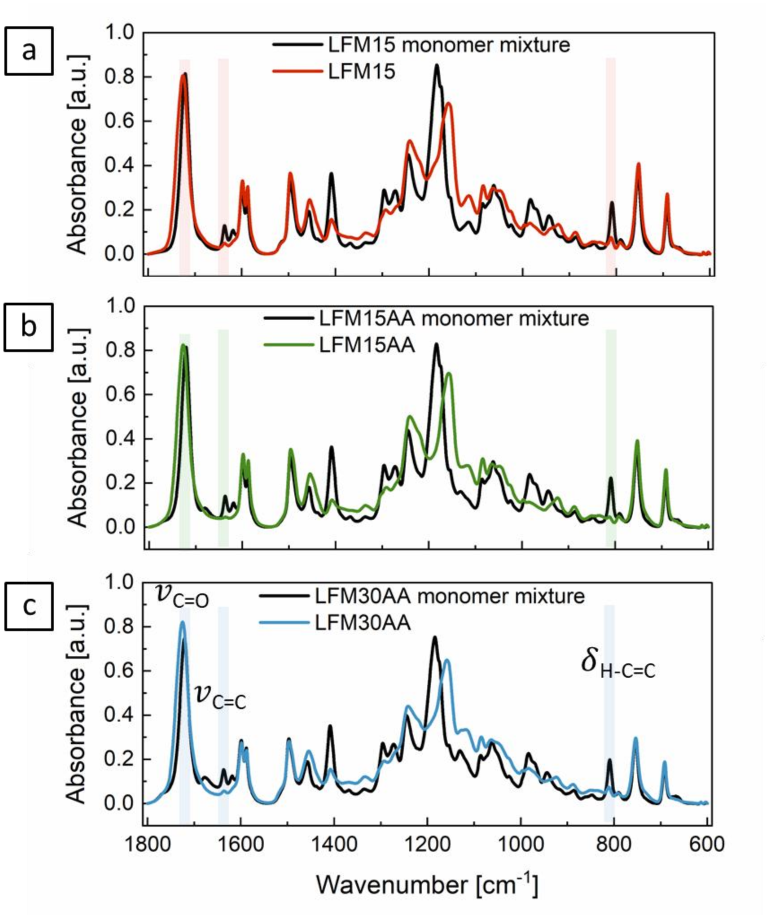

Figure S8. FTIR spectra of the monomer mixtures compared to the DLP 3D-printed components after post-processing via UV-light irradiation for 5 min, and 24 hours in EtOH of a) LFM15, b) LFM15AA, and c) LFM30AA.

Table S13. Determination of residual surface double bonds from FTIR spectra by evaluating the decrease in C=C stretching vibration between the monomer mixture and the 3D-printed components (integral analysis from 1,650 to 1,628 cm^{-1}).

Sample	Residual double bonds [%]
**LFM15**	15.9 ± 0.7
**LFM15AA**	5.3 ± 0.7
**LFM30AA**	14.5 ± 0.7
